# Assessing potential pathogenicity of novel highly pathogenic avian influenza (H5N6) viruses isolated from Mongolian wild duck feces using a mouse model

**DOI:** 10.1080/22221751.2022.2069515

**Published:** 2022-05-25

**Authors:** Bao Tuan Duong, Duc Duong Than, Ulaankhuu Ankhanbaatar, Delgerzul Gombo-Ochir, Gansukh Shura, Amartuvshin Tsolmon, Chris Ka Pun Mok, Ganzorig Basan, Seon Ju Yeo, Hyun Park

**Affiliations:** a Zoonosis Research Center, Department of Infection Biology, School of Medicine, Wonkwang University, Iksan, Korea; b State Central Veterinary Laboratory, Zaisan, Ulaanbaatar, Mongolia; c HKU-Pasteur Research Pole, School of Public Health, Li Ka Shing Faculty of Medicine, The University of Hong Kong, Hong Kong SAR, China; d Department of Tropical Medicine and Parasitology, Department of Biomedical Sciences, Seoul National University College of Medicine, Seoul, Korea

**Keywords:** Avian influenza viruses, Mongolia, H5N6, 2.3.4.4h, hemagglutination inhibition

## Abstract

Several novel highly pathogenic avian influenza (HPAIVs) A(H5N6) viruses were reported in Mongolia in 2020, some of which included host-specific markers associated with mammalian infection. However, their pathogenicity has not yet been investigated. Here, we isolated and evaluate two novel genotypes of A(H5N6) subtype in Mongolia during 2018–2019 (A/wildDuck/MN/H5N6/2018-19). Their evolution pattern and molecular characteristics were evaluated using gene sequencing and their pathogenicity was determined using a mouse model. We also compared their antigenicity with previous H5 Clade 2.3.4.4 human isolates by cross-hemagglutination inhibition (HI). Our data suggests that A/wildDuck/MN/H5N6/2018-19 belongs to clade 2.3.4.4h, and maintains several residues associated with mammal adaptation. In addition, our evaluations revealed that their isolates are less virulent in mice than the previously identified H5 human isolates. However, their antigenicity is distinct from other HPAIVs H5 clade 2.3.4.4, thus supporting their continued evaluation as potential infection risks and the preparation of novel candidate vaccines for their neutralization.

## Introduction

Highly pathogenic avian influenza (HPAI) subtype (H5N1) A/goose/Guangdong/1/1996 virus evolved into 10 genotypes belonging to viral clades (0–9) and multiple subclades [1]. Subclade 2.3.4.4, which includes HPAI A(H5Nx), was first reported in China in 2013 and has since been shown to be globally circulating [2–5].

Wild migratory birds are well known natural reservoirs for the re-assortment and transmission of avian influenza as well as the HPAI H5 viruses from clade 2.3.4.4 [6]. These birds are also known to spread these viruses to geographically distinct regions resulting in panzootic waves of infection killing millions of domestic birds and inducing major economic losses for poultry farmers while increasing their threat to human health [4,7,8]. To date, there have been 72 reported cases of human infections with influenza A (H5N6) virus including 30 deaths [9].

There are two methods of classifying clade 2.3.4.4 H5 HPAI subtype viruses both of which are based on hemagglutinin (HA) protein diversity. Viruses were initially classified into four groups including Group A, B, C, and D [4,10] but recent evaluations have seen this clade getting re-divided by the World Health Organization into eight further subclades (2.3.4.4a–h) [11]. Global surveillance data suggests that there are two dominant H5 HPAI clades 2.3.4.4b and 2.3.4.4h, in circulation across Asia, Europe and the Middle East [7,12–19], with seven human cases of H5N8 and five human cases of H5N6/2.3.4.4b reported in the Russian Federation and China, respectively, in 2021 [20]. Clade 2.3.4.4h viruses were also detected in wild migratory birds and humans in China, Vietnam, Mongolia and Bangladesh [11,15,17,21]. Remarkably, the H5N6/2.3.4.4h A/Chongqing/00013/2021 strain was isolated from a human sample in 2021 and its sequences were uploaded to the GSAID databases with GenBank ID: EPI_ISL_1081369 [20].

Mongolia is located in north-central Asia, and boasts a wide variety of migratory birds. It is widely considered as the most important region for studying the epizootic origins of HPAIVs [22–24]. There were a total of eight H5 HPAIV outbreaks from clades 2.2 and 2.3.2.1, as confirmed by the Mongolian AIV surveillance programme, in Mongolia from 2005 and 2011 and there have been no cases of H5 HPAI reported in this region from 2012 and 2019 [22]. However, there were several HPAI H5N6 2.3.4.4h clade viruses isolated from wild whooper swan in Mongolia in 2020, some of which included mammalian host-specific markers [15,25]. However, the biological characteristics and antigenicity of these viruses have not yet been investigated.

The national surveillance and collaboration programmes for avian influenza viruses (AIVs) funded by the Korean government have facilitated the isolation of many avian influenza subtypes from wild birds, including the two H5N6 HPAIVs isolated from wild duck feces during 2018/2019 in Mongolia [11,26–29]. Here, we focused on the characterization of these two H5N6 isolates and tried to understand the relationship and evolution pattern of these viruses using phylogenetic analysis of each gene segment and the comparison of key mutations with other potential H5 HPAI sequences. We also performed a mouse model study and HI evaluations to determine the pathogenic potential and antigenicity of these viruses.

## Methods

### Sample collection and isolation

Fresh wild bird feces were collected using sterile swabs and stored in liquid nitrogen before transporting to the laboratory. In the laboratory, the feces samples were resuspended in phosphate buffered saline (PBS) supplemented with 100 Unit/µL of penicillin and 100 mg/µL of streptomycin (Gibco, Catalog: 15140122) and were clarified by centrifugation (3000 rpm for 10 min at 4°C), then filtered using a 0.45 µM filter (GVS Syringe, Novatech, USA) before 100 µL of each sample was injected into 9 days-old embryonated specific-pathogen-free (SPF) chicken eggs and incubated at 37°C for 3 days. The allantoic fluids from the eggs were then collected and used to evaluate the viral titre for each isolate using the hemagglutination assay as recommended by the World Organization for Animal Health (http://www.oie.int/eng/normes/mmanual/Asummry).

### Next generation sequencing (NGS) on an Illumina Hiseq X

NGS was completed by GnCBIO (Dae-Jeon, Korea) using the Hiseq X method as previously described [29]. Briefly, the influenza RNA content were extracted using a NUCLEOSPIN® RNA VIRUS (Macherey Nagel) kit and then used as a template for the cDNA library which was evaluated for quality and concentration using LightCycler qPCR. Library size was checked by TapeStation HS D5000 Screen Tape and then subject to NGS.

### Phylogenetic tree analyses

Any related viral genes sequences were identified using the Basic Local Alignment Search Tool (BLAST) from a combined dataset generated using the National Centre for Biotechnology Information (NCBI) and the Global Initiative on Sharing All Influenza Data (GISAID) databases. Phylogenetic trees for each of the eight gene segments (PB2, PB1, PA, NP, HA, NA, M, and NS) were created using MEGA 11 (Molecular Evolutionary Genetics Analysis version 11, Pennsylvania State University, PA, USA), the neighbour joining method, and a maximum composite likelihood model with 1,000 bootstrap replicates used to determine statistical significance (https://www.megasoftware.net/.)

We also attempted to estimate the time of most recent common ancestry (tMRCA), using a time-scaled phylogenitic analysis of the surface viral genes from A/wildDuck/MN/H5N6/2018-19 isolates using the Bayesian Markov chain Monte Carlo method available in BEAST v1.10.4 [30]. Briefly, Multiple sequences were aligned with the BioEdit software (version 7.2.5) for constructing the “non-clock” phylogenetic tree and determining the best substitution model for each dataset by using the IQ-TREE server, then subjected to TempEst analysis [31,32]. We then selected GRT + F + G4 and HKY + F + G4 substitution models and an uncorrelated relaxed clock in combination with a log-normal distribution to evaluate HA and NA datasets, respectively. MCMC chains were run for 50 million generations, with sampling parameters and trees every 5000 generations to produce a relevant post-burn-in effective sample size (ESS) of at least 200 for each parameter using Tracer v1.7.1. The MCC tree was then generated using TreeAnnotator v1.10.4 with 10% burn-in and visualized using FigTree v1.10.4. The tMRCA was calculated as described in the BEAST tutorials (https://taming-the-beast.org/tutorials/Prior-selection/).

### Animal study

We purchased our female six-week-old BALB/c mice from Orient Bio-Seongnam, Gyeonggi, Korea (5 mice per cage) and then infected them with various 50% egg infectious dose (EID_50_) viral concentrations (10^1^ to 10^6^ EID_50_, 50 µL/mouse) using the intranasal infection route. We then evaluated the body weight change and survival rates for each group over 14 days post infection. All of these experiments were carried out in an animal biosafety level 3 (ABSL3) at the Korean Zoonosis Research Institute (KZRI) at Jeonbuk National University. These studies were approved by the KZRI Committee on the Ethics of Animal Experiments (JBNU 2021-0126) and conducted in accordance with all good laboratory practice guidelines.

### Hemagglutination inhibition assay

A/wildDuck/MN/H5N6/2018-19 and other comparable viruses (H5Nx clades 0; 2.3.4.4c; 2.3.4.4e) were inoculated into 10-day-old embryonated chicken eggs for 12 h at 37°C, before the allantoic fluid was harvested for viral inactivation by 0.1% formalin over 16 h at 37°C [33]. Confirmation of virus inactivation through two serial passages in embryonated chicken eggs with no viral RNA detectable by reverse transcription-polymerase chain reaction (rt-PCR) of the matrix gene segment. These viruses were then purified using ultra centrifugation at 112,600*g*, during 2 h, 4°C, and the pellet was re-suspended in PBS before the viral titre was estimated by hemagglutination assay. Chicken antisera products were obtained by immunization with mixtures of 1280 hemagglutination units (HAU) of inactivated virus and an adjuvant (Montanide ISA 70) as described by the manufacturer (https://www.seppic.com/en/montanide-isa-w-o). The chicken blood was then collected 4 weeks after immunization, and then centrifuged at 1200*g* for 10 min at room temperature (20–25°C). The cross reactivity among the four viral subtypes was examined using the specific anti-serum for each virus via the hemagglutinin inhibition (HI) assay as previously described [26,34]. Briefly, the chicken’s antisera were treated with receptor destroying enzyme (Denka Seiken, catalog 370013) to remove nonspecific inhibitors, then serially diluted with PBS into a 96-well plate before 4 hemagglutination units of the appropriate virus was added to the plate and incubated for 30 min at 37°C. Finally, 0.75% chicken red blood was added to each well to complete the HI reaction and the results from the highest serum sample dilution ratio that inhibited hemagglutination after 30 min of incubation were included in our results.

## Results

### Isolation and genetic characteristics

**Figure 1.**
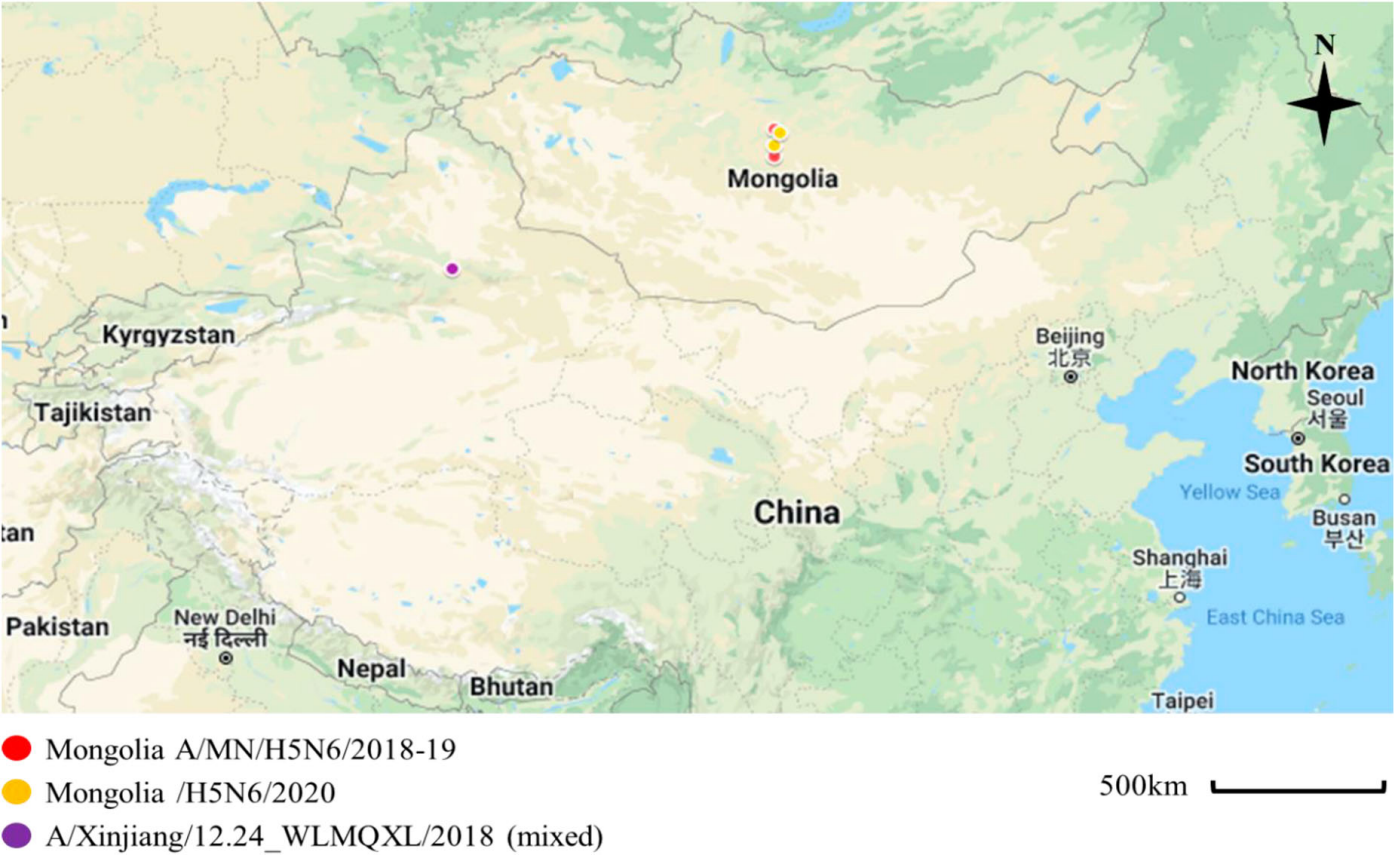
Sampling locations of the Mongolia A/MN/H5N6/2018-19 indicated with red circles (A/duck/Ugii lake/#66/2018/H5N6; A/duck/Khunt lake/#500/2019/H5N6), Mongolia/H5N6/2020 indicated with yellow circles (A/Whooper swan/Mongolia/24/2020/H5N6; A/Swan goose/Mongolia/02/2020/H5N6), and Xinjiang, China viruses indicated with violet circle (A/goose/Xinjiang/12.24_WLMQXL003-C/2018/H5N6-mixed). These sampling sites were close to the site where the novel H5N6/2020 isolates were collected with both sites being separated by about 90 km. Map was edited from Google Maps; March, 15, 2022 (https://www.google.com/maps/d/edit?hl=en&mid=1ZZ4NSJSmmXZo5lbR5GidJWsy422SceyE&ll=28.563846898417626%2C85.17260164694872&z=4).

We isolated 38 AIVs from 2000 fresh wild bird feces samples collected in Mongolia between July 2018 and September 2019 We then used subtype specific polymerase chain reaction (PCR) amplification of the HA and neuraminidase (NA) genes from these samples. Among them, H3N8 subtypes viruses were the predominant subtype (19/38, 50%), and the proportion of other AIVs isolates followed by H4N6 (26.32%), H7N7 (7.89%), H5N6; H2N2 (each 2/28, 5.25%), H1N1; H4N2 (each 1/28, 2.63%) (Appendix Table 1, see Supplemental data). Two H5N6 samples were collected at different lakes; Ugii lake in the Arkhangai Province and Khunt Lake in the Bulgan Province during September 2018 and July 2019, respectively (Figure 1).

We then used NGS to facilitate the identification of the 16 complete viral segments comprising the genomes of the A/duck/Ugii lake/#66/2018(H5N6) and A/duck/Khunt lake/#500/2019(H5N6) isolates. These sequences were then annotated using the Influenza virus sequence annotation tool (https://www.ncbi.nlm.nih.gov/genomes/FLU/annotation/) and submitted to the NCBI database under GenBank IDs MW822900 ∼ MW822915. Both the NGS and BLAST results of the full-length sequences against the NCBI and GSAID databases revealed that both A/MN/H5N6/2018-19 viruses were identical across all eight gene segments (PB2, PB1, PA, NP, HA, NA, M, and NS), with a high degree of common nucleotide identity with A/Xinjiang/12.24_WLMQXL/2018 (mixed) like viruses (99.74%–100%) (Table 1). However, these viruses shared low nucleotide identity with Mongolia H5N6 isolated in 2020 (97.46%–98.73%) (Appendix Table 2, see Supplemental data). Given this information, we further evaluated the likely origins of these viruses using maximum-likelihood phylogenetic trees produced via the neighbour-joining method in MEGAX. All eight gene segments of the HPAI A/wildDuck/MN/H5N6/2018-19 viruses clustered with the same segments from the China A/goose/Xinjiang/12.24_WLMQXL/2018 (Mixed)-like viruses which have not been investigated before, but we know that these viruses belong to H5 HPAIV clade 2.3.4.4h (Group C) (Appendix Figure 1, see Supplemental data). Taken together, these data suggest that the MG/H5N6/2018-19 viruses have extensive similarity with the A/Xinjiang/12.24_WLMQXL/2018 like viruses.

**Table 1.** Closest relatives to our viral nucleotide sequences from A/MN/H5N6/2018-19 in the GenBank databases and GISAID.^a^

Gene segment	Closest relative	Homology	GenBank ID
PB2	A/chicken/Xinjiang/12.24_WLMQXL006-O/2018 (mixed)	99.74%	MW106970.1
	A/chicken/Guangdong/7.20_DGCP022-O/2017	99.08%	MW104088.1
PB1	A/goose/Xinjiang/12.24_WLMQXL004-O/2018 (mixed)	99.82%	MW110193.1
	A/chicken/Guangdong/7.20_DGCP022-O/2017 (mixed)	99.74%	MW104088.1
PA	A/goose/Xinjiang/12.24_WLMQXL004-C/2018 (mixed)	99.77%	MW110193.1
	A/chicken/Guangdong/7.20_DGCP022-O/2017 (A/H0)	99.49%	MW104088.1
HA	A/goose/Xinjiang/12.24_WLMQXL003-C/2018 (mixed)	99.65%	MW110187.1
	A/chicken/Guangdong/7.20_DGCP022-O/2017 (mixed)	98.71%	MW104088.1
NP	A/goose/Xinjiang/12.24_WLMQXL004-O/2018 (mixed)	99.80%	MW110193.1
	A/chicken/Guangdong/7.20_DGCP022-O/2017 (mixed)	99.47%	MW104088.1
NA	A/chicken/Xinjiang/12.24_WLMQXL006-O/2018 (mixed)	99.78%	MW106970.1
	/duck/Hunan/11.30_YYGK62E3-OC/2017 (A/H5N6)	99.06%	MW108138.1
M	A/goose/Xinjiang/12.24_WLMQXL003-C/2018 (mixed)	100.00%	MW110187.1
	A/duck/Guangdong/7.20_DGCP036-O/2017(H5N6)	99.74%	MW097501.1
NS	A/goose/Xinjiang/12.24_WLMQXL004-O/2018 (mixed)	99.85%	MW110196.1
	A/chicken/Guangdong/7.20_DGCP022-O/2017(mixed)	99.56%	MW104088.1

^a^ Last accessed 12 November 2021 NCBI, https://www.ncbi.nlm.nih.gov/Structure/cdd/wrpsb.cgi; GISAID, https://platform.epicov.org/epi3/frontend#384e22. HA, hemagglutinin; NA, neuraminidase; MP, matrix protein; NP, nucleoprotein; NS, nonstructural protein; PA, acidic polymerase; PB1, basic polymerase 1; PB2, basic polymerase 2.

**Figure 2.**
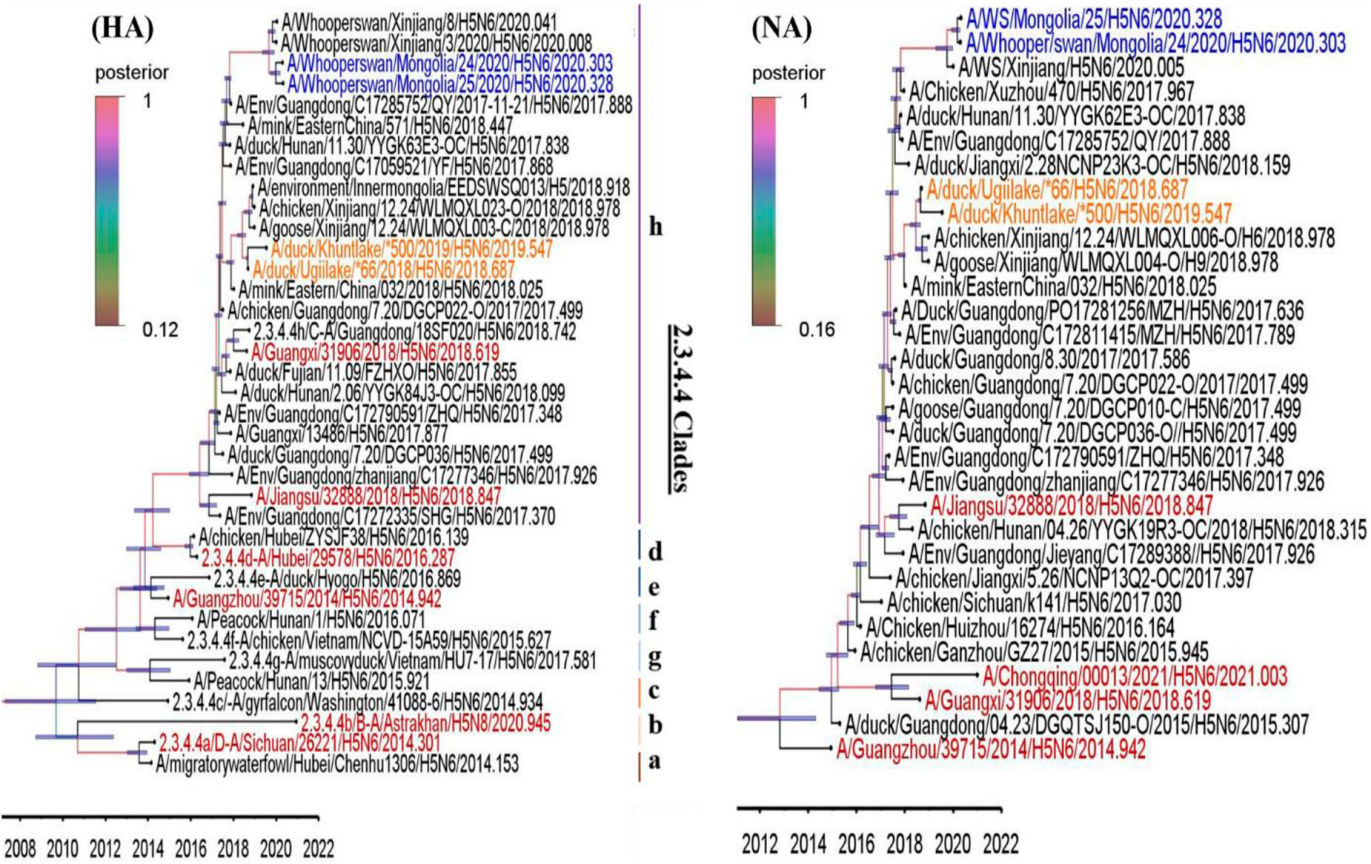
The HA and NA gene maximum clade credibility tree for the A/MN/H5N6/2018-19 viruses was constructed using the BEAST 1.10.4 software package (https://beast-dev.github.io/beast-mcmc). Node bars indicate a 95% posterior density for each node height and the coloured of each branch indicate its posterior probability. Both H5N6 isolates reported in this study are shown in orange, the human H5 in red and the MN/H5N6/2020 in blue. The tMRCA is indicated at the bottom of the tree, and was estimated using the Bayesian Markov chain Monte Carlo method in BEAST 1.10.4.

We then estimated the tMRCA for the surface gene segments in these viruses using a relaxed Bayesian clock phylogenetic analysis in BEAST v1.10.4. tMRCA for both the HA and NA genes of the A/wildDuck/MN/H5N6/2018-19 viruses suggest that they and the A/Xinjiang/12.24_WLMQXL/2018 viruses evolved from a common ancestor which was likely in circulation in China in May to Jul 2018 (Figure 2; Table 2) while, the tMRCA for the HA and NA genes of the A/wildDuck/MN/H5N6/2018-19 viruses ranged from July to September 2018 suggest its reached Mongolia during summer season.

**Table 2.** Date of isolation for the most recent common ancestor for the HPAIVs H5N6 Mongolia/ 2018–2019 and Xinjiang/2018 viruses when using the surface gene as the comparator.

Gene segment	tMRCA^a^ Mean (Moth & Year)	tMRCA 95% HPD^#^ (Moth & Year)	Posterior probability
*A/wildDuck/MN/H5N6/2018-19 and A/Xinjiang/12.24_WLMQXL/2018 viruses*			
HA	MAY.2018	MAR.2018 – JUL.2018	1
NA	JUL.2018	MAR.2018 – AUG.2018	0.99
*A/wildDuck/MN/H5N6/2018-19*			
HA	SEP.2018	JUL.2018 – SEP.2018	1
NA	AUG.2018	JUL.2018 – SEP.2018	0.99

^a^ tMRCA, time of most recent common ancestry. tMRCA represents the most likely timing for a common ancestral node; ^#^HPD, highest posterior density; HA, hemagglutinin; NA, neuraminidase; Posterior probability suggest a high corresponding confidence measure for phylogenetic accuracy when calculated as a value close to 1.

### Molecular characteristics

We then went on to assess the potential virulence of the A/wildDuck/MN/H5N6/2018-19 viruses by comparing their amino acid sequences with those from known H5 HPAI 2.3.4.4 h clade viruses including the 2020 Mongolian isolates (MG/H5N6/2020) and the 5 human isolates identified between 2018 and 2021 (Table 3). We found the characteristic HA cleavage site polybasic residues (RERRRK(K)R/G) in seven of these isolates including the A/wildDuck/MN/H5N6/2018-19; A/Whooper Swan/Mongolia/24/2020; A/Chongqing/00013/2021; A/Guangzhou/39715/2014; A/Guangxi/31906/2018; A/Jiangsu/32888/2018 and A/Hong Kong/483/1997, which is considered to be the signature of virulence marker for HPAI viruses [35]. Additionally, these viruses had several mutations in the HA receptor-binding sites associated with increased affinity for the *α*-2,6 linked sialic acid (SA) human-like receptor including 94N, 133A, and 156A [36–38]. Most of these viruses also maintained the characteristic amino acid residues at position 222Q, 223R and 224G (H5 numbering) associated with specificity for the avian-like α-2,3 SA receptor [39,40]. The amino acid deletions of NA at 58 to 68 and nonstructural protein 1 (NS1) at 80 to 84 positions are both associated with high pathogenicity in avian hosts [41,42]. However, two mutations in PB2, including 627K and 701N, which are known to contribute to increased virulence in mammalian models, were not detected in our A/wildDuck/MN/H5N6/2018-19 isolates and A/Chongqing/00013/2021(H5N6) human isolates. In general, the mutation in HA of A/wildDuck/MN/H5N6/2018-19 isolates that change receptor binding specificity suggests that these viruses are potentially virulent for mammals.

**Table 3.** Molecular characteristics of the HPAI A/MN/H5N6/2018-19 isolates identified in this study.

Virus	Host	Clade 2.3.4.4	PB2		PB1-F2	HA^a^							NA^b^	NS
			627	701	Expression	Cleavage site	94	133	156	222	223	224	Stalk deletion 58–68	Deletion 80–84
1	Duck	h	E	D	NO	RERRRKR↓G	N	A	A	Q	R	G	Yes	Yes
2	WS	h	E	D	NO	RERRRKR↓G	N	A	A	Q	R	G	Yes	Yes
3	Human	h	E	D	NO	RERRRKR↓G	N	A	A	Q	R	G	Yes	Yes
4	Human	h	K	D	NO	RERRRKR↓G	N	A	A	Q	R	G	Yes	Yes
5	Human	h	K	D	NO	RERRRKR↓G	N	–	A	Q	G	G	Yes	Yes
6	Human	e	K	D	NO	RERRRKR↓G	N	A	T	Q	R	G	Yes	Yes
7	Human	clade 0	K	D	NO	RERRRKKR↓G	N	S	T	Q	S	G	Yes	No

^a^ H5, numbering.

^b^ N6, numbering; WS, Whooper swan; 1, HPAI MG/H5N6/2018-19 isolates; 2, A/Whooper Swan/Mongolia/24/2020 (H5N6); 3, A/Chongqing/00013/2021(H5N6); 4, A/Guangxi/31906/2018 (H5N6); 5, A/Jiangsu/32888/2018 (H5N6); 6, A/Guangzhou/39715/2014 (H5N6); 7, A/Hong Kong/483/1997 (H5N1).

### Pathogenic potential in a mouse model of infection

**Figure 3.**
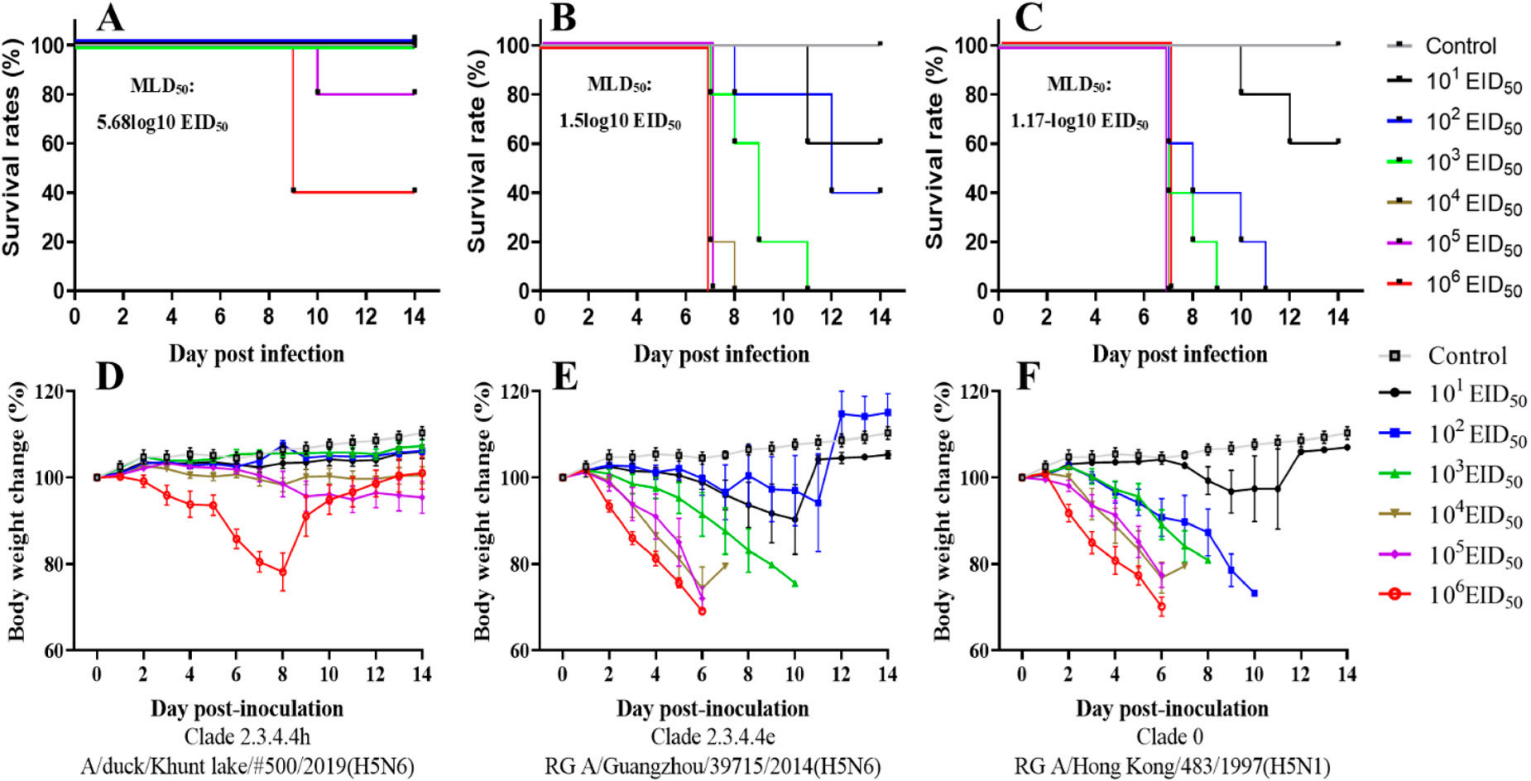
Comparison of H5N6 Mongolia 2019 isolate pathogenicity and Human isolate pathogenicity in a mouse model. Each group of five 6-week-old mice were inoculated via the intranasal route with between 10^1^ and 10^6^ EID_50_ per mouse for each virus in each mouse and then evaluated for survival and body weight change over a 14 day period DPI. A-D, Subclade 2.3.4.4 h A/duck/Khunt lake/#500/2019(H5N6); B-E, Subclade 2.3.4.4e RG A/Guangzhou/39715/2014 (H5N6); C-F, Clade 0 RG A/Hong Kong/483/1997 (H5N1).

Given these characteristics, we then assessed the pathogenicity of the A/wildDuck/MN/H5N6/2018-19 viruses using the intranasal inoculation of six-week-old mice with 10^1^ to 10^6^ EID_50_ per mouse of MG/H5N6/2019 and two human isolates, A/Hong Kong/483/1997 (H5N1) from clade 0 and A/Guangzhou/39715/2014 (H5N6) from clade 2.3.4.4e (Figure 3). Both body weight and survival rate were monitored over a 14- day post infection (DPI) period for each group with these values then used to determine the fifty percent mouse lethal dose (MLD_50_) for each strain. The mice infected with the A/wildDuck/MN/H5N6/2019 virus exhibited only minor pathogenic effect, with survival rates between 80% and 100% at 14 DPI when infected with 10^1^ to 10^5^ EID_50_ per mouse via intranasal inoculation. However, an increase in the viral dose to 10^6^ EID_50_ per mouse resulted in a decrease in survival to 40% and a concomitant 25% decrease in body weight at 8 DPI (MLD_50_; 5.68-log10 EID_50_). Meanwhile, both the human H5N6 and H5N1 isolates killed 100% of the mice with each presenting with severe clinical signs of more than 25% weight loss within 7 DPI when infected with between 10^3^ and 10^6^ EID_50_ per mouse. This data produces very low MLD_50_ values for both H5N1 (MLD_50_; 1.17-log10 EID_50_) and H5N6 (MLD_50_; 1.5-log10 EID_50_), suggesting that MG/H5N6/2018-19 viruses have low pathogenicity in mice, especially when compared with human origin isolates.

### Antigenic characterization

To understand the antigenic profiles of the A/wildDuck/MN/H5N6/2018-19 subclade 2.3.4.4h isolates, we performed HI assays by using antisera generated in SPF chickens against H5 HPAIs clades 0; 2.3.4.4e; 2.3.4.4c and 2.3.4.4h MG/H5N6/2019. The results showed that the HI titres of antisera against the homologous clades 0; 2.3.4.4e; 2.3.4.4c and 2.3.4.4h viruses were ≥2048, 1024, 1024, and ≥2048, respectively (Table 4). The A/wildDuck/MN/H5N6/2019 subclade 2.3.4.4h virus and clade 0 responded poorly to antiserum against each other, with subclades 2.3.4.4e; 2.3.4.4c suffering a drop of 16 to 128-folds HI titre units when compared to homologous titres, whereas the clade 2.3.4.4e antiserum cross-reacted with the subclade 2.3.4.4c viruses with HI titres that were 2-fold lower than that to the homologous virus. The results of HI assays revealed that A/wildDuck/MN/H5N6/2019 subclade 2.3.4.4h isolates are antigenically different from the two H5 HPAIs human isolates from subclades 2.3.4.4e, 2.3.4.4c, and clade 0, although the subclades 2.3.4.4e and 2.3.4.4c antigenicity is comparable.

**Table 4.** HI evaluations of HPAI A(H5N6)/Mongolia /2019 influenza viruses using chicken antiserum.

Clade	HA inhibition titre (dilution factor) in antiserum against clades			
	0	2.3.4.4e	2.3.4.4c	2.3.4.4h
0	**≥2048**	≤16	64	64
2.3.4.4e	128	**1024**	512	128
2.3.4.4c	128	512	**1024**	16
2.3.4.4h	128	32	128	**≥2048**

HA, Hemagglutinin. Homologous titres are boldface and underlined

Clade 0: A (H5N1)/Hong Kong/483/1997; subclade 2.3.4.4e: A(H5N6)/Guangzhou/39715/2014.

Subclade 2.3.4.4c: A (H5N6)/Duck/Foshan/41/2019; subclade 2.3.4.4h: A(H5N6)/Mongolia /2019.

## Discussion

Three features that make Mongolia a unique location for studying the epizootiology of HPAIV in wild bird populations. Firstly, millions of migratory birds breed and molt across Mongolia as it stretches between their arctic grounds and their wintering sites in the south [43]. Secondly, the country has little domestic poultry, making transmission of these avian influenzas the sole provenance of the wild birds [22]. Third, at least eight different flyways across Mongolia have been shown to be used by migratory birds [44]. Interestingly, Mongolia is located within three major flyways for various wild birds, including the East Asian–Australasian, Central Asian, and East African–West Asian flyways, which overlap with an additional four flyways including the Black Sea, East Atlantic, Pacific American, and Mississippi American routes, meaning that Mongolia’s wild migratory birds might carry these viruses into these cross sections and spread them throughout the world [45]. Therefore, continual monitoring and surveillance of the wild birds in Mongolia is necessary to control AIVs as well as H5 HPAI outbreaks in the future.

Recent years have seen the replacement of the H5N1 subtype with H5N6 and H5N8 HPAIVs from the clade 2.3.4.4 as the dominant subtypes in circulation [14,16,21,46,47]. Here, we collected two new H5N6 isolates from nearby sites (90 km away from each other) that demonstrate a high degree of nucleotide similarity. Our phylogenetic analysis demonstrated that A/MN/H5N6/2018-19 belongs to subclade 2.3.4.4h, with all eight gene segments showing high degrees of identity with the (A) Xinjiang/12.24_WLMQXL/2018 like viruses (99.74%–100%). However, seven of these gene segments shared significantly lower degrees of nucleotide identity with the novel Mongolian H5N6 isolates reported in 2020 (97.46%–98.73%), the one gene that exhibits any significant similarity (99.21%) was the M gene (Appendix Table 2, see Supplemental data) [15,25]. These results demonstrate that our novel A/wildDuck/MN/H5N6/2018-19 and the H5N6 Mongolia 2020 isolates form two distinct genotypes.

During winter (November to January), migratory birds stay in their wintering grounds and then move northwards between February and May (spring migration) to reach their nesting grounds for the new season, they will stay in these northern regions until the temperatures begin to drop and they are forced to return South to their wintering grounds with this migration taking place between August and November [48]. These migration patterns and our tMRCA data of A/wildDuck/MN/H5N6/2018-19 (July to September 2018) hypothesize that these migratory waterfowl pick up H5N6 viruses during their spring migration, and carry it to their breeding ground in Mongolia where they stay between May and August. This was consistent with a previous study that suggested that the spring bird migration pattern matched with the timing and route of H5N6 virus transmission between Xinjiang and Mongolia [15]. Taken together, this data suggests that the spring migration plays a significant role in the transmission and circulation of HPAI H5N6 in Mongolia.

The monobasic cleavage motif PQRETR↓GLF in HA is digested by trypsin-like proteases in the upper respiratory tract of various avian species and is common in less pathogenic (LP) AIVs, while HPAIVs carry a poly-basic amino acid motif RERRRKR↓G which is recognized by furin, a commonly expressed mammalian enzyme that allows for rapid replication in mammalian cells [29,49]. Our data suggests that the HA gene from A/wildDuck/MN/H5N6/2018-19 encodes the RERRRKR↓G poly-basic cleavage motif with a high degree of similarity to four H5 HPAI isolates of human origin including A/Chongqing/00013/2021, A/Guangxi/31906/2018, and A/Jiangsu/32888/2018 (Table 3). However, the transition of LP viruses to HPAIVs requires not only the addition of this polybasic HA cleavage site but also adaptive changes in neighbouring regions or in the other viral proteins that facilitate mammalian replication [50,51]. Thus, other site mutations in the HA biding site like those associated with increased *α*-2,6 linked sialic acid (SA) human-like receptor affinity such as, 94N, 133A, and 156A may contribute to the increased virulence observed in our A/wildDuck/MN/H5N6/2018-19 isolates. In addition, our A/MN/H5N6/2018-19 viruses also contained several amino acid mutations associated with increased virulence in avian species including both the deletion of specific residues in the NA stalk and the NS1 gene. The NA stalk deletion has been reported to act as a major virulence factor for chicken infections [42], while the 80 to 84 residue deletion in the NS1 gene has been linked to the increased pathogenicity of H5N1 AIVs in mallard ducks [41]. Interestingly, mammalian adaptive markers in PB2, including 627 K, were not detected in both of A/Chongqing/00013/2021 and A/wildDuck/MN/H5N6/2018-19, 2020 viruses, this suggests that even without 627 K biomarker, some of these viruses can infect to human. It is also worth noting that the 701N mutation was not found in any of the H5 viruses evaluated in this study [52]. Seven of the isolates encoded the PB1-F2 truncation, which likely contributes to their viral pathogenicity [53]. In general, the key amino acid sequences in the A/wildDuck/MN/H5N6/2018-19 isolates were similar to those of the A/Chongqing/00013/2021 human isolate suggesting their potential threat to public health. Given this, we suggest that further pathogenicity evaluations in other models should be used to determine the true threat of human infection with A/wildDuck/MN/H5N6/2018-19 viruses.

A(H5N1)/HongKong/483/1997 and A(H5N6)/Guangzhou/39715/2014 isolated from a hospitalized patient both demonstrated high virulence and severe lethality in BALB/c mice at a very low MLD_50_ value [54,55]. This was consistent with our results were the MLD_50_ values for A(H5N1)/HongKong/483/1997 and A(H5N6)/Guangzhou/39715/2014 were 1.17-log10 EID_50_ and 1.5-log10 EID_50_, respectively. Meanwhile, our evaluations of the A/wildDuck/MN/H5N6/2018-19 isolate demonstrated that it had low pathogenicity in mice, with an MLD_50_ value of 5.68-log10 EID_50_, which was significantly higher than that of either human isolate. This result does corroborate our molecular characterization which showed that the A/wildDuck/MN/H5N6/2018-19 isolate lacked two of the individual mutations in the PB2 protein (lysine at position 627 to glutamic acid and aspartate at position 701 to asparagine) leading to low pathogenicity in mice [52,56]. However, the A/wildDuck/MN/H5N6/2018-19 isolate MLD_50_ value (5.68-log10 EID50) was lower than the MLD_50_ (6.38-log_10_ EID_50_) of the A (H5N6) Xinjiang/2020/2.3.4.4 h virus identified in China in 2020, despite its close relationship with the recently described 2020 H5N6 Mongolian isolates [15,17]. This suggests that the potential virulence of the A/wildDuck/MN/H5N6/2018-19 isolates are likely to be higher than the 2020 H5N6 Mongolian isolates.

The antigenic characterization assay described by the Centres for Disease Control and Prevention (CDC) suggest that influenza viruses are likely to be antigenically dissimilar if their HI titres vary by five or more fold (https://www.cdc.gov/flu/about/professionals/antigenic.htm). Our HI results were highly consistent with previous studies, indicating that H5 HPAIVs from the clade 2.3.4.4 exhibit considerable antigenic variation [57–59]. Both A/wildDuck/MN/H5N6/2018-19 HPAIVs from subclade 2.3.4.4h were antigenically distinct from H5 HPAIVs from clades 0 and 2.3.4.4e and c, suggesting the development of candidate vaccines for H5 HPAIVs subclade 2.3.4.4h are needed. This result was also consistent with the results of the molecular analysis of the HA genes from these isolates which revealed significant differences in the amino acid sequences of their receptor binding sites with a series of mutations including S133A, T156A, and R223N (Table 3) described in each.

In conclusion, animal model studying and antigenic characterization have broadened our understanding of the HPAIV subclade 2.3.4.4h, with our data suggesting that the H5N6 viruses isolated in 2018–2019 have low pathogenicity in mice and that these isolates were also antigenically dissimilar from other H5 clade 2.3.4.4 isolates from human hosts. These results also highlight the probability of transmission and spread of H5 HPAIVs to Mongolia via wild migratory birds during their spring migration, further supporting the ongoing surveillance of these viruses in Mongolia. In addition, this data demonstrates that both surveillance and the identification of new antigenic subtypes should be supported to facilitate the development of novel vaccine candidates that could be urgently implemented to control the global spread of H5 HPAIVs preventing both economic loss and reducing the public health risks associated with potential exposure.

## Supplementary Material

Supplemental Material

## References

[1] Verhagen JH, Munster VJ, Fouchier RAM. Ecology and evolution of avian influenza viruses. In: M Tibayrenc, editor. Genetics and evolution of infectious disease. London: Elsevier; 2011. p. 729–749.

[2] Lee DH. Evolution, global spread, and pathogenicity of highly pathogenic avian influenza H5Nx clade 2.3.4.4. J Vet Sci. 2017;18(S1):269–280. 28859267 10.4142/jvs.2017.18.S1.269 PMC5583414

[3] Qi X. Whole-genome sequence of a Reassortant H5N6 Avian influenza virus isolated from a Live Poultry Market in China, 2013. Genome Announ. 2014;2:5. 10.1128/genomeA.00706-14 PMC4161740 25212611

[4] Shin J. Highly pathogenic H5N6 avian influenza virus subtype clade 2.3.4.4 indigenous in South Korea. Sci Rep. 2020;10(1):7241. 32350323 10.1038/s41598-020-64125-x PMC7190616

[5] Zhang J. Genetic diversity, phylogeography, and evolutionary dynamics of highly pathogenic avian influenza A (H5N6) viruses. Virus Evol. 2020;6(2):veaa079. 33324491 10.1093/ve/veaa079 PMC7724252

[6] Tian H. Avian influenza H5N1 viral and bird migration networks in Asia. Proc Natl Acad Sci U S A. 2015;112(1):172–177. 25535385 10.1073/pnas.1405216112 PMC4291667

[7] Pyankova OG. Isolation of clade 2.3.4.4b A(H5N8), a highly pathogenic avian influenza virus, from a worker during an outbreak on a poultry farm, Russia, December 2020. Euro Surveill. 2021;26:24. 10.2807/1560-7917.ES.2021.26.24.2100439 PMC8212591 34142650

[8] Poen MJ. Co-circulation of genetically distinct highly pathogenic avian influenza A clade 2.3.4.4 (H5N6) viruses in wild waterfowl and poultry in Europe and East Asia, 2017–18. Virus Evol. 2019;5(1):vez004. 31024736 10.1093/ve/vez004 PMC6476160

[9] WHO. Avian influenza weekly update number 832. https://www.who.int/docs/default-source/wpro—documents/emergency/surveillance/avian-influenza/ai_20220218.pdf?sfvrsn=5f006f99_87.

[10] Lee DH. Highly pathogenic avian influenza viruses and generation of novel reassortants, United States, 2014–2015. Emerg Infect Dis. 2016;22(7):1283–1285. 27314845 10.3201/eid2207.160048 PMC4918163

[11] Trinh TT. Genetic characterization and pathogenesis of avian influenza virus H7N3 isolated from spot-billed ducks in South Korea, early 2019. Viruses. 2021;13:5. 10.3390/v13050856 PMC8151380 34067187

[12] Zecchin B. Evolutionary dynamics of H5 highly pathogenic avian influenza viruses (clade 2.3.4.4B) circulating in Bulgaria in 2019–2021. Viruses. 2021;13:10. 10.3390/v13102086 PMC8541051 34696516

[13] Sobolev I. Highly pathogenic avian influenza a(H5N8) virus clade 2.3.4.4b, Western Siberia, Russia, 2020. Emerg Infect Dis. 2021;27(8):2224–2227. 34287138 10.3201/eid2708.204969 PMC8314819

[14] Liang Y. Novel clade 2.3.4.4b highly pathogenic avian influenza A H5N8 and H5N5 viruses in Denmark, 2020. Viruses. 2021;13:5. 10.3390/v13050886 PMC8151437 34065033

[15] Jeong S. Highly pathogenic avian influenza clade 2.3.4.4 subtype H5N6 viruses isolated from wild Whooper swans, Mongolia, 2020. Emerg Infect Dis. 2021;27(4):1181–1183. 33754986 10.3201/eid2704.203859 PMC8007304

[16] Ali M. Genetic characterization of highly pathogenic avian influenza A(H5N8) virus in Pakistani live bird markets reveals rapid diversification of clade 2.3.4.4b viruses. Viruses. 2021;13: 8. 10.3390/v13081633 PMC8402709 34452498

[17] Li Y. Outbreaks of highly pathogenic avian influenza (H5N6) virus subclade 2.3.4.4h in Swans, Xinjiang, Western China, 2020. Emerg Infect Dis. 2020;26(12):2956–2960. 33030424 10.3201/eid2612.201201 PMC7706961

[18] Jeong S. Highly pathogenic avian influenza clade 2.3.4.4b subtype H5N8 virus isolated from Mandarin Duck in South Korea, 2020. Viruses. 2020;12:12. 10.3390/v12121389 PMC7761861 33291548

[19] Selim AA. Highly pathogenic avian influenza virus (H5N8) clade 2.3.4.4 infection in migratory birds, Egypt. Emerg Infect Dis. 2017;23(6):1048–1051. 28518040 10.3201/eid2306.162056 PMC5443452

[20] Xiao C. Five independent cases of human infection with avian influenza H5N6 – Sichuan province, China, 2021. China CDC Wkly. 2021;3(36):751–756. 34594983 10.46234/ccdcw2021.187 PMC8427102

[21] Turner JCM. Highly pathogenic avian influenza A(H5N6) virus clade 2.3.4.4h in wild birds and live poultry markets, Bangladesh. Emerg Infect Dis. 2021;27(9):2492–2494. 34424167 10.3201/eid2709.210819 PMC8386775

[22] Gilbert M. Highly pathogenic avian influenza virus among wild birds in Mongolia. PLoS One. 2012;7(9):e44097. 22984464 10.1371/journal.pone.0044097 PMC3439473

[23] Kang HM. Genetic analyses of avian influenza viruses in Mongolia, 2007 to 2009, and their relationships with Korean isolates from domestic poultry and wild birds. Poult Sci. 2011;90(10):2229–2242. 21934005 10.3382/ps.2011-01524

[24] Spackman E. Characterization of low pathogenicity avian influenza viruses isolated from wild birds in Mongolia 2005 through 2007. Virol J. 2009;6:190. 19891786 10.1186/1743-422X-6-190 PMC2781007

[25] Ankhanbaatar U. Isolation and identification of a highly pathogenic avian influenza H5N6 virus from migratory waterfowl in Western Mongolia. J Wildl Dis. 2021;58(1):211–214. 10.7589/JWD-D-21-00032 34699593

[26] Yeo SJ. Emergence of a novel reassortant H5N3 avian influenza virus in Korean mallard ducks in 2018. Intervirology. 2021;65(1):1–16. 34438407 10.1159/000517057 PMC8820440

[27] Trinh TT. Emergence of novel reassortant H1N1 avian influenza viruses in Korean wild ducks in 2018 and 2019. Viruses. 2020;13:1. 33375376 10.3390/v13010030 PMC7823676

[28] Nguyen NM. Genetic characterization of a novel north American-origin avian influenza A (H6N5) virus isolated from bean goose of South Korea in 2018. Viruses. 2020;12:7. 10.3390/v12070774 PMC7411716 32709116

[29] Yeo SJ. Molecular characterization of a novel avian influenza A (H2N9) strain isolated from wild duck in Korea in 2018. Viruses. 2019;11:11. 10.3390/v11111046 PMC6893532 31717636

[30] Suchard MA. Bayesian phylogenetic and phylodynamic data integration using BEAST 1.10. Virus Evol. 2018;4(1):vey016. 29942656 10.1093/ve/vey016 PMC6007674

[31] Trifinopoulos J. W-IQ-TREE: a fast online phylogenetic tool for maximum likelihood analysis. Nucleic Acids Res. 2016;44(W1):W232–W235. 27084950 10.1093/nar/gkw256 PMC4987875

[32] Rambaut A. Exploring the temporal structure of heterochronous sequences using TempEst (formerly Path-O-Gen). Virus Evol. 2016;2(1):vew007. 27774300 10.1093/ve/vew007 PMC4989882

[33] Pawar SD. Evaluation of different inactivation methods for high and low pathogenic avian influenza viruses in egg-fluids for antigen preparation. J Virol Methods. 2015;222:28–33. 25997377 10.1016/j.jviromet.2015.05.004

[34] Kaufmann L. An optimized hemagglutination inhibition (HI) assay to quantify influenza-specific antibody titers. J Vis Exp. 2017;130:55833. 10.3791/55833 PMC5755515 29286466

[35] Luczo JM. Evolution of high pathogenicity of H5 avian influenza virus: haemagglutinin cleavage site selection of reverse-genetics mutants during passage in chickens. Sci Rep. 2018;8(1):11518. 30068964 10.1038/s41598-018-29944-z PMC6070550

[36] Su Y. Analysis of a point mutation in H5N1 avian influenza virus hemagglutinin in relation to virus entry into live mammalian cells. Arch Virol. 2008;153(12):2253–2261. 19020946 10.1007/s00705-008-0255-y

[37] Yang ZY. Immunization by avian H5 influenza hemagglutinin mutants with altered receptor binding specificity. Science. 2007;317(5839):825–828. 17690300 10.1126/science.1135165 PMC2367145

[38] Herfst S. Airborne transmission of influenza A/H5N1 virus between ferrets. Science. 2012;336(6088):1534–1541. 22723413 10.1126/science.1213362 PMC4810786

[39] Gaide N. Pathobiology of highly pathogenic H5 avian influenza viruses in naturally infected Galliformes and Anseriformes in France during winter 2015–2016. Vet Res. 2022;53(1):11. 35164866 10.1186/s13567-022-01028-x PMC8842868

[40] Stevens J. Structure and receptor specificity of the hemagglutinin from an H5N1 influenza virus. Science. 2006;312(5772):404–410. 16543414 10.1126/science.1124513

[41] Li Y. A 20-amino-acid deletion in the neuraminidase stalk and a five-amino-acid deletion in the NS1 protein both contribute to the pathogenicity of H5N1 avian influenza viruses in mallard ducks. PLoS One. 2014;9(4):e95539. 24743258 10.1371/journal.pone.0095539 PMC3990698

[42] Stech O. The neuraminidase stalk deletion serves as major virulence determinant of H5N1 highly pathogenic avian influenza viruses in chicken. Sci Rep. 2015;5:13493. 26306544 10.1038/srep13493 PMC4549673

[43] Wildlife Science and Conservation Center of Mongolia. http://www.wscc.org.mn/p/11.

[44] The East Asian-Australasian Flyway Partnership. https://www.eaaflyway.net/the-flyway/.

[45] Ulaankhuu A. Genetic and antigenic characterization of H5 and H7 avian influenza viruses isolated from migratory waterfowl in Mongolia from 2017 to 2019. Virus Genes. 2020;56(4):472–479. 32430568 10.1007/s11262-020-01764-2 PMC7235438

[46] Li H. Continuous reassortment of clade 2.3.4.4 H5N6 highly pathogenetic avian influenza viruses demonstrating high risk to public health. Pathogens. 2020;9:8. 10.3390/pathogens9080670 PMC7460007 32824873

[47] Abolnik C. Outbreaks of clade 2.3.4.4 H5N8 highly pathogenic avian influenza in 2018 in the northern regions of South Africa were unrelated to those of 2017. Transbound Emerg Dis. 2020;67(3):1371–1381. 31833671 10.1111/tbed.13448

[48] Zhang J. Determination of original infection source of H7N9 avian influenza by dynamical model. Sci Rep. 2014;4:4846. 24786135 10.1038/srep04846 PMC5381286

[49] Bogs J. Highly pathogenic H5N1 influenza viruses carry virulence determinants beyond the polybasic hemagglutinin cleavage site. PLoS One. 2010;5(7):e11826. 20676399 10.1371/journal.pone.0011826 PMC2910732

[50] Gohrbandt S. Amino acids adjacent to the haemagglutinin cleavage site are relevant for virulence of avian influenza viruses of subtype H5. J Gen Virol. 2011;92(Pt 1):51–59. 20881092 10.1099/vir.0.023887-0

[51] Stech O. Acquisition of a polybasic hemagglutinin cleavage site by a low-pathogenic avian influenza virus is not sufficient for immediate transformation into a highly pathogenic strain. J Virol. 2009;83(11):5864–5868. 19297482 10.1128/JVI.02649-08 PMC2681970

[52] Zhu W. Dual E627K and D701N mutations in the PB2 protein of A(H7N9) influenza virus increased its virulence in mammalian models. Sci Rep. 2015;5:14170. 26391278 10.1038/srep14170 PMC4585756

[53] Kamal RP. Emergence of highly pathogenic avian influenza A(H5N1) virus PB1-F2 variants and their virulence in BALB/c mice. J Virol. 2015;89(11):5835–5846. 25787281 10.1128/JVI.03137-14 PMC4442455

[54] Spesock A. The virulence of 1997 H5N1 influenza viruses in the mouse model is increased by correcting a defect in their NS1 proteins. J Virol. 2011;85(14):7048–7058. 21593152 10.1128/JVI.00417-11 PMC3126612

[55] Pan W. Patient-derived avian influenza A (H5N6) virus is highly pathogenic in mice but can be effectively treated by anti-influenza polyclonal antibodies. Emerg Microbes Infect. 2018;7(1):107. 29899428 10.1038/s41426-018-0113-2 PMC6000000

[56] Salomon R. The polymerase complex genes contribute to the high virulence of the human H5N1 influenza virus isolate A/Vietnam/1203/04. J Exp Med. 2006;203(3):689–697. 16533883 10.1084/jem.20051938 PMC2118237

[57] Ilyicheva T. Antibodies to highly pathogenic A/H5Nx (clade 2.3.4.4) influenza viruses in the sera of Vietnamese residents. Pathogens. 2021;10:4. 10.3390/pathogens10040394 PMC8064466 33806156

[58] Herfst S. Human clade 2.3.4.4 A/H5N6 influenza virus lacks mammalian adaptation markers and does not transmit via the airborne route between ferrets. mSphere. 2018;3:1. 10.1128/mSphere.00405-17 PMC5750386 29299528

[59] Ohkawara A. Antigenic diversity of H5 highly pathogenic avian influenza viruses of clade 2.3.4.4 isolated in Asia. Microbiol Immunol. 2017;61(5):149–158. 28370432 10.1111/1348-0421.12478

