# Supplementary material for: Assessing potential pathogenicity of novel highly pathogenic avian influenza (H5N6) viruses isolated from Mongolian wild duck feces using a mouse model
Source: Emerg Microbes Infect. 2022 May 25;11(1):1425–34. doi: 10.1080/22221751.2022.2069515 (PMC9154755; doi:10.1080/22221751.2022.2069515)
Supplement: Supplemental Material [file TEMI_A_2069515_SM9821.pdf]

Appendix table 1: List of IAVs isolated during July 2018 and September 2019 in Mongolia.

No.	Time of Sample Collection	Sample Type	Location (Province)	Subtype (HA)	Proportion[a] (%)
1	2019-July	Feces	Bulgan	H1N1	2.63
2	2018-Sep	Feces	Doit Lake	H2N2	5.26
3	2018-Sep	Feces	Khunt lake	H2N2	
4	2018-Sep	Feces	Arkhangai	H3N8	50.00
5	2018-Sep	Feces	Arkhangai	H3N8	
6	2018-Sep	Feces	Arkhangai	H3N8	
7	2018-Sep	Feces	Arkhangai	H3N8	
8	2018-Sep	Feces	Doit Lake	H3N8	
9	2018-Sep	Feces	Doit Lake	H3N8	
10	2018-Sep	Feces	Doit Lake	H3N8	
11	2018-Sep	Feces	Doit Lake	H3N8	
12	2018-Sep	Feces	Doit Lake	H3N8	
13	2018-Sep	Feces	Khunt lake	H3N8	
14	2019-May	Feces	Zavkhan	H3N8	
15	2019-May	Feces	Zavkhan	H3N8	
16	2019-May	Feces	Zavkhan	H3N8	
17	2019-May	Feces	Zavkhan	H3N8	
18	2019-July	Feces	Bulgan	H3N8	
19	2019-July	Feces	Bulgan	H3N8	
20	2019-July	Feces	Bulgan	H3N8	
21	2019-July	Feces	Bulgan	H3N8	
22	2019-July	Feces	Bulgan	H3N8	
23	2019-May	Feces	Khuvsgul	H4N2	2.63
24	2018-Sep	Feces	Arkhangai	H4N6	26.32
25	2018-Sep	Feces	Arkhangai	H4N6	
26	2018-Sep	Feces	Arkhangai	H4N6	
27	2018-Sep	Feces	Khunt lake	H4N6	
28	2018-Sep	Feces	Khunt lake	H4N6	
29	2019-May	Feces	Zavkhan	H4N6	
30	2019-May	Feces	Zavkhan	H4N6	
31	2019-July	Feces	Bulgan	H4N6	
32	2019-July	Feces	Bulgan	H4N6	
33	2019-July	Feces	Bulgan	H4N6	
34	2018-Sep	Feces	Arkhangai	H5N6	5.26
35	2019-July	Feces	Bulgan	H5N6	
36	2019-May	Feces	Khuvsgul	H7N7	7.89
37	2019-May	Feces	Khuvsgul	H7N7	
38	2019-May	Feces	Khuvsgul	H7N7	

[a][(Number of each subtype viruses)/(number of total AIV-subtype viruses)] × 100 (%).

Appendix Table 2. Comparisons of the close relatives of HPAI (H5N6) Mongolia 2018-2019 and (H5N6) Mongolia 2020 isolates.

A/MN/H5N6/2018-19	A/MN/H5N6/2020*	Homology	Segment ID
PB2	A/Whooper swan/Mongolia/24/2020	97.50%	EPI_ISL_707015
	A/Swan goose/Mongolia/02/2020(H5N6)	97.59%	MW690022.1
PB1	A/Whooper swan/Mongolia/24/2020	98.73%	EPI_ISL_707015
	A/Swan goose/Mongolia/02/2020(H5N6)	-	No data in NCBI
PA	A/Whooper swan/Mongolia/24/2020	98.96%	EPI_ISL_707015
	A/Swan goose/Mongolia/02/2020(H5N6)	98.88%	MW689683.1
HA	A/Whooper swan/Mongolia/24/2020	97.46%	EPI_ISL_707015
	A/Swan goose/Mongolia/02/2020(H5N6)	97.65%	MW689547.1
NP	A/Whooper swan/Mongolia/24/2020	98.05%	EPI_ISL_707015
	A/Swan goose/Mongolia/02/2020(H5N6)	97.86%	MW689632.1
NA	A/Whooper swan/Mongolia/24/2020	98.33%	EPI_ISL_707015
	A/Swan goose/Mongolia/02/2020(H5N6)	98.26%	MW692371.1
M	A/Whooper swan/Mongolia/24/2020	99.01%	EPI_ISL_707015
	A/Swan goose/Mongolia/02/2020(H5N6)	99.21%	MW689618.1
NS	A/Whooper swan/Mongolia/24/2020	98.42%	EPI_ISL_707015
	A/Swan goose/Mongolia/02/2020(H5N6)	98.53%	MW689629.1

A (PB2)

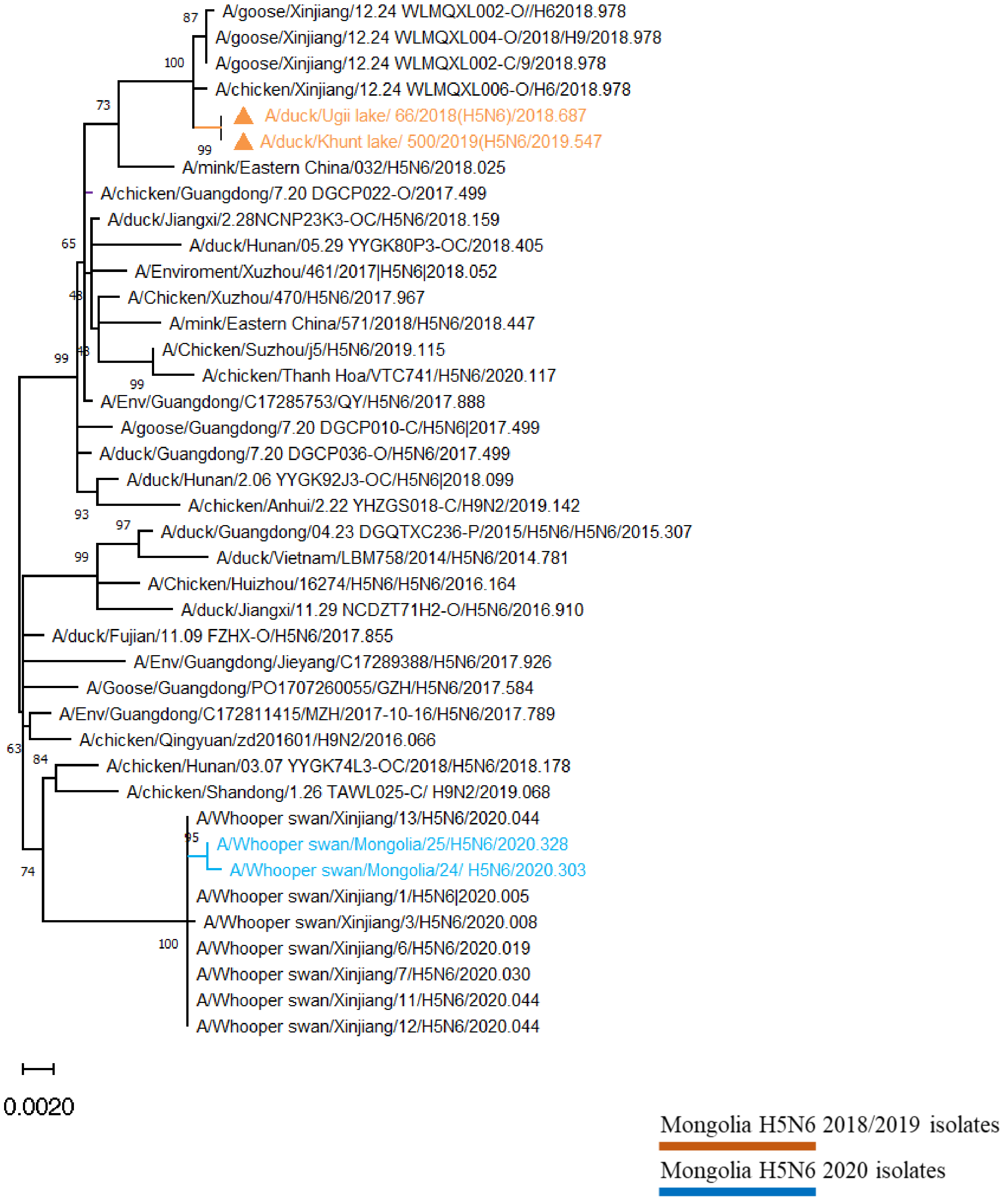
A/goose/Xinjiang/12.24 WLMQXL002-O//H62018.978
A/goose/Xinjiang/12.24 WLMQXL004-O/2018/H9/2018.978
A/goose/Xinjiang/12.24 WLMQXL002-C/9/2018.978
A/chicken/Xinjiang/12.24 WLMQXL006-O/H6/2018.978
A/duck/Ugii lake/ 66/2018(H5N6)/2018.687
A/duck/Khunt lake/ 500/2019(H5N6/2019.547
A/mink/Eastern China/032/H5N6/2018.025
A/chicken/Guangdong/7.20 DGCP022-O/2017.499
A/duck/Jiangxi/2.28NCNP23K3-OC/H5N6/2018.159
A/duck/Hunan/05.29 YYGK80P3-OC/2018.405
A/Enviroment/Xuzhou/461/2017|H5N6|2018.052
A/Chicken/Xuzhou/470/H5N6/2017.967
A/mink/Eastern China/571/2018/H5N6/2018.447
A/Chicken/Suzhou/j5/H5N6/2019.115
A/chicken/Thanh Hoa/VTC741/H5N6/2020.117
A/Env/Guangdong/C17285753/QY/H5N6/2017.888
A/goose/Guangdong/7.20 DGCP010-C/H5N6|2017.499
A/duck/Guangdong/7.20 DGCP036-O/H5N6/2017.499
A/duck/Hunan/2.06 YYGK92J3-OC/H5N6|2018.099
A/chicken/Anhui/2.22 YHZGS018-C/H9N2/2019.142
A/duck/Guangdong/04.23 DGQTXC236-P/2015/H5N6/H5N6/2015.307
A/duck/Vietnam/LBM758/2014/H5N6/2014.781
A/Chicken/Huizhou/16274/H5N6/H5N6/2016.164
A/duck/Jiangxi/11.29 NCDZT71H2-O/H5N6/2016.910
A/duck/Fujian/11.09 FZHX-O/H5N6/2017.855
A/Env/Guangdong/Jieyang/C17289388/H5N6/2017.926
A/Goose/Guangdong/PO1707260055/GZH/H5N6/2017.584
A/Env/Guangdong/C172811415/MZH/2017-10-16/H5N6/2017.789
A/chicken/Qingyuan/zd201601/H9N2/2016.066
A/chicken/Hunan/03.07 YYGK74L3-OC/2018/H5N6/2018.178
A/chicken/Shandong/1.26 TAWL025-C/ H9N2/2019.068
A/Whooper swan/Xinjiang/13/H5N6/2020.044
A/Whooper swan/Mongolia/25/H5N6/2020.328
A/Whooper swan/Mongolia/24/ H5N6/2020.303
A/Whooper swan/Xinjiang/1/H5N6|2020.005
A/Whooper swan/Xinjiang/3/H5N6/2020.008
A/Whooper swan/Xinjiang/6/H5N6/2020.019
A/Whooper swan/Xinjiang/7/H5N6/2020.030
A/Whooper swan/Xinjiang/11/H5N6/2020.044
A/Whooper swan/Xinjiang/12/H5N6/2020.044
87
100
73
99
65
48
48
99
99
93
97
99
63
84
95
74
100
0.0020
Mongolia H5N6 2018/2019 isolates
Mongolia H5N6 2020 isolates

B (PB1)

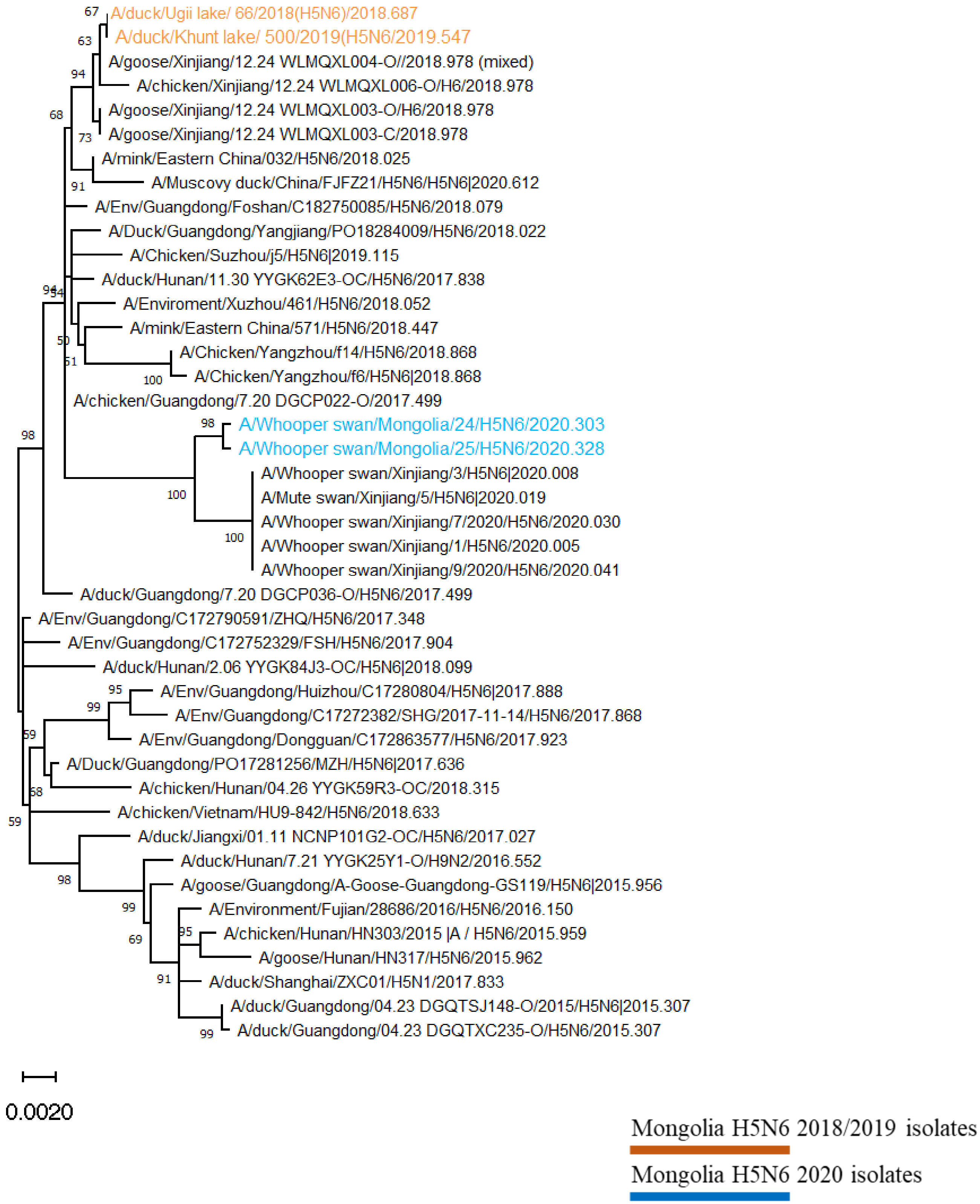
67 A/duck/Ugii lake/ 66/2018(H5N6)/2018.687
63 A/duck/Khunt lake/ 500/2019(H5N6/2019.547
94 A/goose/Xinjiang/12.24 WLMQXL004-O//2018.978 (mixed)
A/chicken/Xinjiang/12.24 WLMQXL006-O/H6/2018.978
68 A/goose/Xinjiang/12.24 WLMQXL003-O/H6/2018.978
73 A/goose/Xinjiang/12.24 WLMQXL003-C/2018.978
A/mink/Eastern China/032/H5N6/2018.025
91 A/Muscovy duck/China/FJFZ21/H5N6/H5N6|2020.612
A/Env/Guangdong/Foshan/C182750085/H5N6/2018.079
A/Duck/Guangdong/Yangjiang/PO18284009/H5N6/2018.022
A/Chicken/Suzhou/j5/H5N6|2019.115
A/duck/Hunan/11.30 YYGK62E3-OC/H5N6/2017.838
94 54 A/Enviroment/Xuzhou/461/H5N6/2018.052
50 A/mink/Eastern China/571/H5N6/2018.447
51 A/Chicken/Yangzhou/f14/H5N6/2018.868
100 A/Chicken/Yangzhou/f6/H5N6|2018.868
A/chicken/Guangdong/7.20 DGCP022-O/2017.499
98 A/Whooper swan/Mongolia/24/H5N6/2020.303
98 A/Whooper swan/Mongolia/25/H5N6/2020.328
A/Whooper swan/Xinjiang/3/H5N6|2020.008
100 A/Mute swan/Xinjiang/5/H5N6|2020.019
A/Whooper swan/Xinjiang/7/2020/H5N6/2020.030
100 A/Whooper swan/Xinjiang/1/H5N6/2020.005
A/Whooper swan/Xinjiang/9/2020/H5N6/2020.041
A/duck/Guangdong/7.20 DGCP036-O/H5N6/2017.499
A/Env/Guangdong/C172790591/ZHQ/H5N6/2017.348
A/Env/Guangdong/C172752329/FSH/H5N6/2017.904
A/duck/Hunan/2.06 YYGK84J3-OC/H5N6|2018.099
95 A/Env/Guangdong/Huizhou/C17280804/H5N6|2017.888
99 A/Env/Guangdong/C17272382/SHG/2017-11-14/H5N6/2017.868
59 A/Env/Guangdong/Dongguan/C172863577/H5N6/2017.923
A/Duck/Guangdong/PO17281256/MZH/H5N6|2017.636
68 A/chicken/Hunan/04.26 YYGK59R3-OC/2018.315
59 A/chicken/Vietnam/HU9-842/H5N6/2018.633
A/duck/Jiangxi/01.11 NCNP101G2-OC/H5N6/2017.027
98 A/duck/Hunan/7.21 YYGK25Y1-O/H9N2/2016.552
99 A/goose/Guangdong/A-Goose-Guangdong-GS119/H5N6|2015.956
A/Environment/Fujian/28686/2016/H5N6/2016.150
69 95 A/chicken/Hunan/HN303/2015 |A / H5N6/2015.959
A/goose/Hunan/HN317/H5N6/2015.962
91 A/duck/Shanghai/ZXC01/H5N1/2017.833
A/duck/Guangdong/04.23 DGQTSJ148-O/2015/H5N6|2015.307
99 A/duck/Guangdong/04.23 DGQTXC235-O/H5N6/2015.307
0.0020
Mongolia H5N6 2018/2019 isolates
Mongolia H5N6 2020 isolates

C (PA)

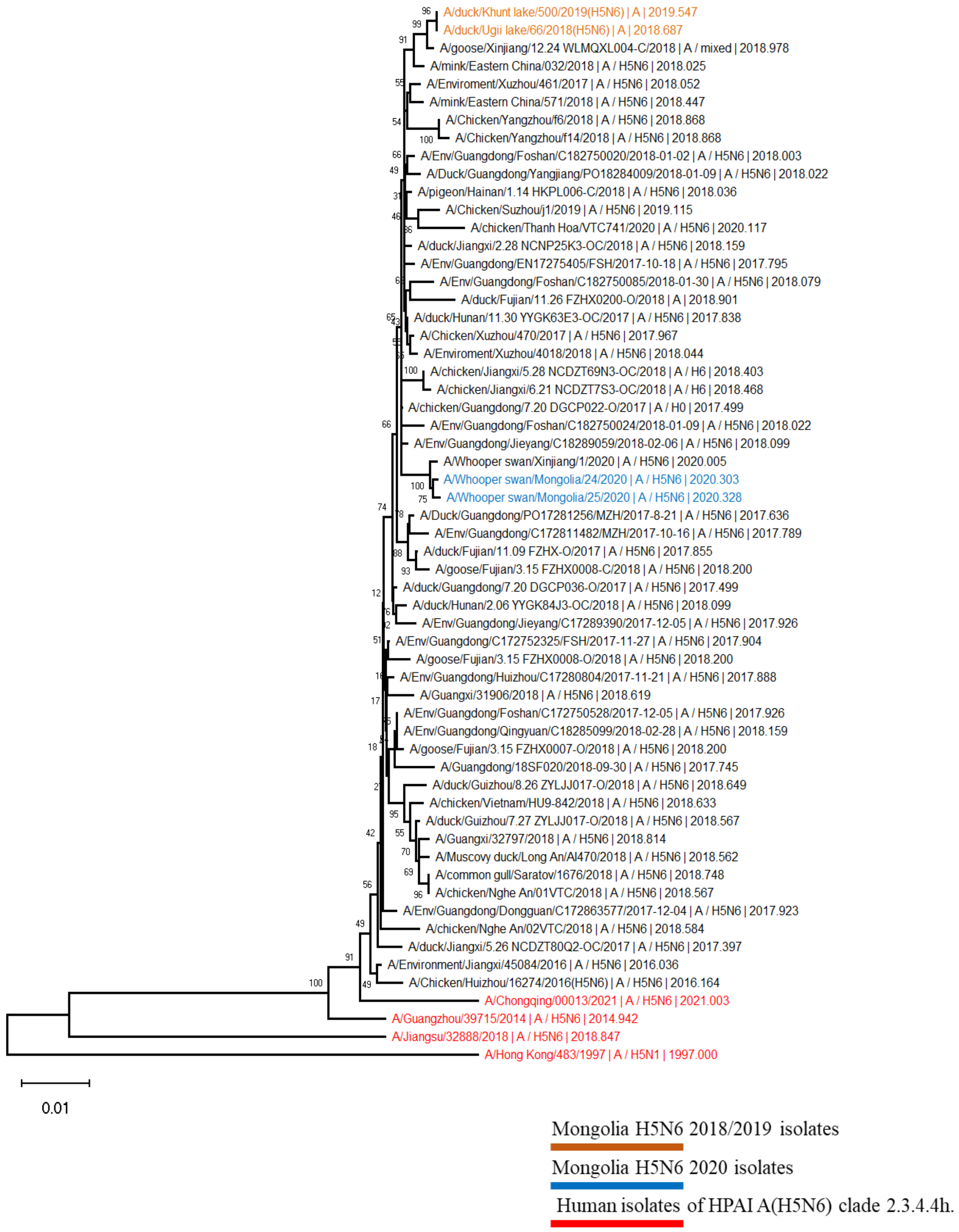
A/duck/Khunt lake/500/2019(H5N6) | A | 2019.547
A/duck/Ugii lake/66/2018(H5N6) | A | 2018.687
A/goose/Xinjiang/12.24 WLMQXL004-C/2018 | A / mixed | 2018.978
A/mink/Eastern China/032/2018 | A / H5N6 | 2018.025
A/Enviroment/Xuzhou/461/2017 | A / H5N6 | 2018.052
A/mink/Eastern China/571/2018 | A / H5N6 | 2018.447
A/Chicken/Yangzhou/f6/2018 | A / H5N6 | 2018.868
A/Chicken/Yangzhou/f14/2018 | A / H5N6 | 2018.868
A/Env/Guangdong/Foshan/C182750020/2018-01-02 | A / H5N6 | 2018.003
A/Duck/Guangdong/Yangjiang/PO18284009/2018-01-09 | A / H5N6 | 2018.022
A/pigeon/Hainan/1.14 HKPL006-C/2018 | A / H5N6 | 2018.036
A/Chicken/Suzhou/j1/2019 | A / H5N6 | 2019.115
A/chicken/Thanh Hoa/VTC741/2020 | A / H5N6 | 2020.117
A/duck/Jiangxi/2.28 NCNP25K3-OC/2018 | A / H5N6 | 2018.159
A/Env/Guangdong/EN17275405/FSH/2017-10-18 | A / H5N6 | 2017.795
A/Env/Guangdong/Foshan/C182750085/2018-01-30 | A / H5N6 | 2018.079
A/duck/Fujian/11.26 FZHX0200-O/2018 | A | 2018.901
A/duck/Hunan/11.30 YYGK63E3-OC/2017 | A / H5N6 | 2017.838
A/Chicken/Xuzhou/470/2017 | A / H5N6 | 2017.967
A/Enviroment/Xuzhou/4018/2018 | A / H5N6 | 2018.044
A/chicken/Jiangxi/5.28 NCDZT69N3-OC/2018 | A / H6 | 2018.403
A/chicken/Jiangxi/6.21 NCDZT7S3-OC/2018 | A / H6 | 2018.468
A/chicken/Guangdong/7.20 DGCP022-O/2017 | A / H0 | 2017.499
A/Env/Guangdong/Foshan/C182750024/2018-01-09 | A / H5N6 | 2018.022
A/Env/Guangdong/Jieyang/C18289059/2018-02-06 | A / H5N6 | 2018.099
A/Whooper swan/Xinjiang/1/2020 | A / H5N6 | 2020.005
A/Whooper swan/Mongolia/24/2020 | A / H5N6 | 2020.303
A/Whooper swan/Mongolia/25/2020 | A / H5N6 | 2020.328
A/Duck/Guangdong/PO17281256/MZH/2017-8-21 | A / H5N6 | 2017.636
A/Env/Guangdong/C172811482/MZH/2017-10-16 | A / H5N6 | 2017.789
A/duck/Fujian/11.09 FZHX-O/2017 | A / H5N6 | 2017.855
A/goose/Fujian/3.15 FZHX0008-C/2018 | A / H5N6 | 2018.200
A/duck/Guangdong/7.20 DGCP036-O/2017 | A / H5N6 | 2017.499
A/duck/Hunan/2.06 YYGK84J3-OC/2018 | A / H5N6 | 2018.099
A/Env/Guangdong/Jieyang/C17289390/2017-12-05 | A / H5N6 | 2017.926
A/Env/Guangdong/C172752325/FSH/2017-11-27 | A / H5N6 | 2017.904
A/goose/Fujian/3.15 FZHX0008-O/2018 | A / H5N6 | 2018.200
A/Env/Guangdong/Huizhou/C17280804/2017-11-21 | A / H5N6 | 2017.888
A/Guangxi/31906/2018 | A / H5N6 | 2018.619
A/Env/Guangdong/Foshan/C172750528/2017-12-05 | A / H5N6 | 2017.926
A/Env/Guangdong/Qingyuan/C18285099/2018-02-28 | A / H5N6 | 2018.159
A/goose/Fujian/3.15 FZHX0007-O/2018 | A / H5N6 | 2018.200
A/Guangdong/18SF020/2018-09-30 | A / H5N6 | 2017.745
A/duck/Guizhou/8.26 ZYLJJ017-O/2018 | A / H5N6 | 2018.649
A/chicken/Vietnam/HU9-842/2018 | A / H5N6 | 2018.633
A/duck/Guizhou/7.27 ZYLJJ017-O/2018 | A / H5N6 | 2018.567
A/Guangxi/32797/2018 | A / H5N6 | 2018.814
A/Muscovy duck/Long An/AI470/2018 | A / H5N6 | 2018.562
A/common gull/Saratov/1676/2018 | A / H5N6 | 2018.748
A/chicken/Nghe An/01VTC/2018 | A / H5N6 | 2018.567
A/Env/Guangdong/Dongguan/C172863577/2017-12-04 | A / H5N6 | 2017.923
A/chicken/Nghe An/02VTC/2018 | A / H5N6 | 2018.584
A/duck/Jiangxi/5.26 NCDZT80Q2-OC/2017 | A / H5N6 | 2017.397
A/Environment/Jiangxi/45084/2016 | A / H5N6 | 2016.036
A/Chicken/Huizhou/16274/2016(H5N6) | A / H5N6 | 2016.164
A/Chongqing/00013/2021 | A / H5N6 | 2021.003
A/Guangzhou/39715/2014 | A / H5N6 | 2014.942
A/Jiangsu/32888/2018 | A / H5N6 | 2018.847
A/Hong Kong/483/1997 | A / H5N1 | 1997.000
0.01
Mongolia H5N6 2018/2019 isolates
Mongolia H5N6 2020 isolates
Human isolates of HPAI A(H5N6) clade 2.3.4.4h.

D (HA)

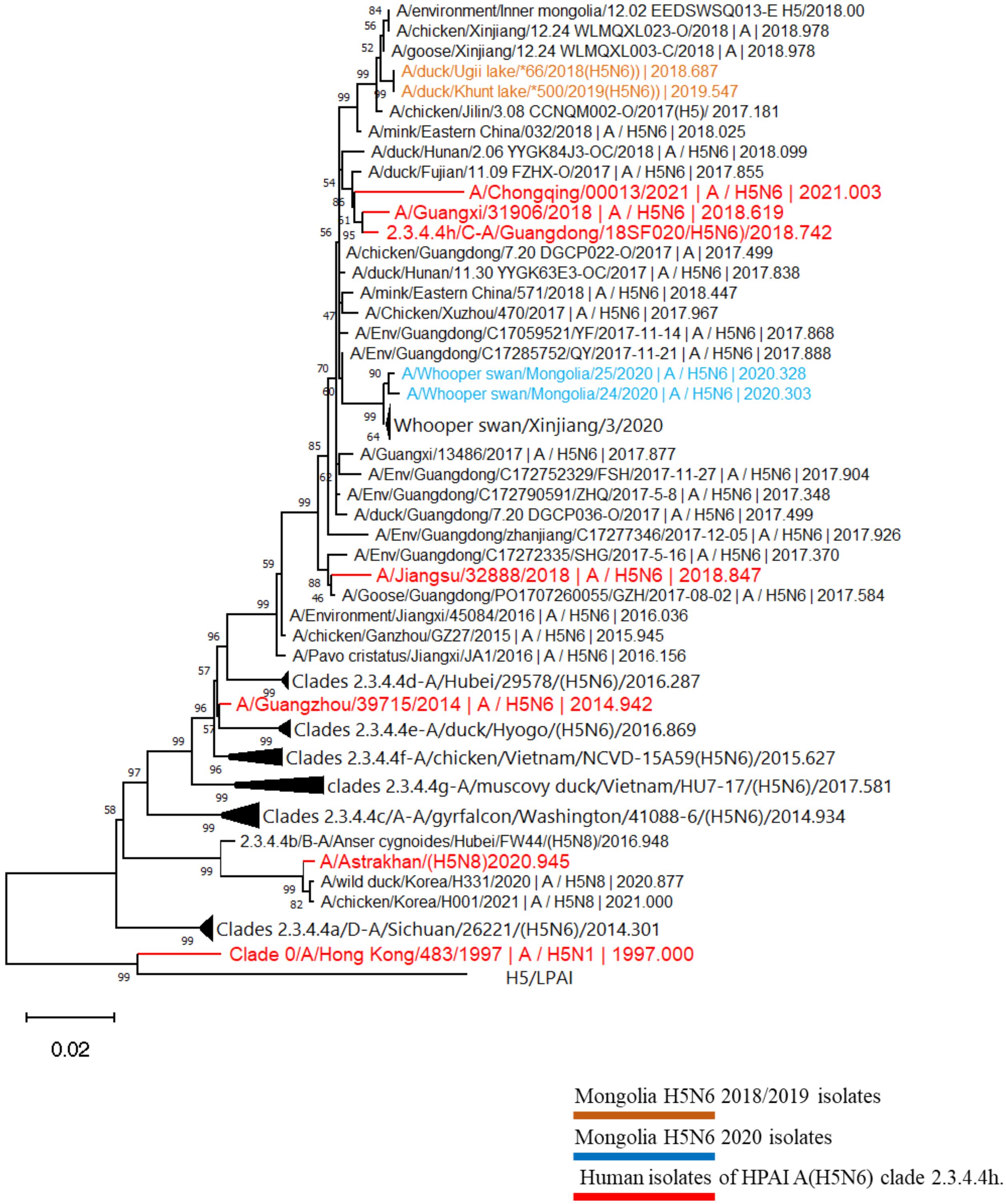
A/environment/Inner mongolia/12.02 EEDSWSQ013-E H5/2018.00
A/chicken/Xinjiang/12.24 WLMQXL023-O/2018 | A | 2018.978
A/goose/Xinjiang/12.24 WLMQXL003-C/2018 | A | 2018.978
A/duck/Ugii lake/*66/2018(H5N6)) | 2018.687
A/duck/Khunt lake/*500/2019(H5N6)) | 2019.547
A/chicken/Jilin/3.08 CCNQM002-O/2017(H5)/ 2017.181
A/mink/Eastern China/032/2018 | A / H5N6 | 2018.025
A/duck/Hunan/2.06 YYGK84J3-OC/2018 | A / H5N6 | 2018.099
A/duck/Fujian/11.09 FZHX-O/2017 | A / H5N6 | 2017.855
A/Chongqing/00013/2021 | A / H5N6 | 2021.003
A/Guangxi/31906/2018 | A / H5N6 | 2018.619
2.3.4.4h/C-A/Guangdong/18SF020/H5N6)/2018.742
A/chicken/Guangdong/7.20 DGCP022-O/2017 | A | 2017.499
A/duck/Hunan/11.30 YYGK63E3-OC/2017 | A / H5N6 | 2017.838
A/mink/Eastern China/571/2018 | A / H5N6 | 2018.447
A/Chicken/Xuzhou/470/2017 | A / H5N6 | 2017.967
A/Env/Guangdong/C17059521/YF/2017-11-14 | A / H5N6 | 2017.868
A/Env/Guangdong/C17285752/QY/2017-11-21 | A / H5N6 | 2017.888
A/Whooper swan/Mongolia/25/2020 | A / H5N6 | 2020.328
A/Whooper swan/Mongolia/24/2020 | A / H5N6 | 2020.303
Whooper swan/Xinjiang/3/2020
A/Guangxi/13486/2017 | A / H5N6 | 2017.877
A/Env/Guangdong/C172752329/FSH/2017-11-27 | A / H5N6 | 2017.904
A/Env/Guangdong/C172790591/ZHQ/2017-5-8 | A / H5N6 | 2017.348
A/duck/Guangdong/7.20 DGCP036-O/2017 | A / H5N6 | 2017.499
A/Env/Guangdong/zhanjiang/C17277346/2017-12-05 | A / H5N6 | 2017.926
A/Env/Guangdong/C17272335/SHG/2017-5-16 | A / H5N6 | 2017.370
A/Jiangsu/32888/2018 | A / H5N6 | 2018.847
A/Goose/Guangdong/PO1707260055/GZH/2017-08-02 | A / H5N6 | 2017.584
A/Environment/Jiangxi/45084/2016 | A / H5N6 | 2016.036
A/chicken/Ganzhou/GZ27/2015 | A / H5N6 | 2015.945
A/Pavo cristatus/Jiangxi/JA1/2016 | A / H5N6 | 2016.156
Clades 2.3.4.4d-A/Hubei/29578/(H5N6)/2016.287
A/Guangzhou/39715/2014 | A / H5N6 | 2014.942
Clades 2.3.4.4e-A/duck/Hyogo/(H5N6)/2016.869
Clades 2.3.4.4f-A/chicken/Vietnam/NCVD-15A59(H5N6)/2015.627
clades 2.3.4.4g-A/muscovy duck/Vietnam/HU7-17/(H5N6)/2017.581
Clades 2.3.4.4c/A-A/gyrfalcon/Washington/41088-6/(H5N6)/2014.934
2.3.4.4b/B-A/Anser cygnoides/Hubei/FW44/(H5N8)/2016.948
A/Astrakhan/(H5N8)2020.945
A/wild duck/Korea/H331/2020 | A / H5N8 | 2020.877
A/chicken/Korea/H001/2021 | A / H5N8 | 2021.000
Clades 2.3.4.4a/D-A/Sichuan/26221/(H5N6)/2014.301
Clade 0/A/Hong Kong/483/1997 | A / H5N1 | 1997.000
H5/LPAI
0.02
Mongolia H5N6 2018/2019 isolates
Mongolia H5N6 2020 isolates
Human isolates of HPAI A(H5N6) clade 2.3.4.4h.

E (NP)

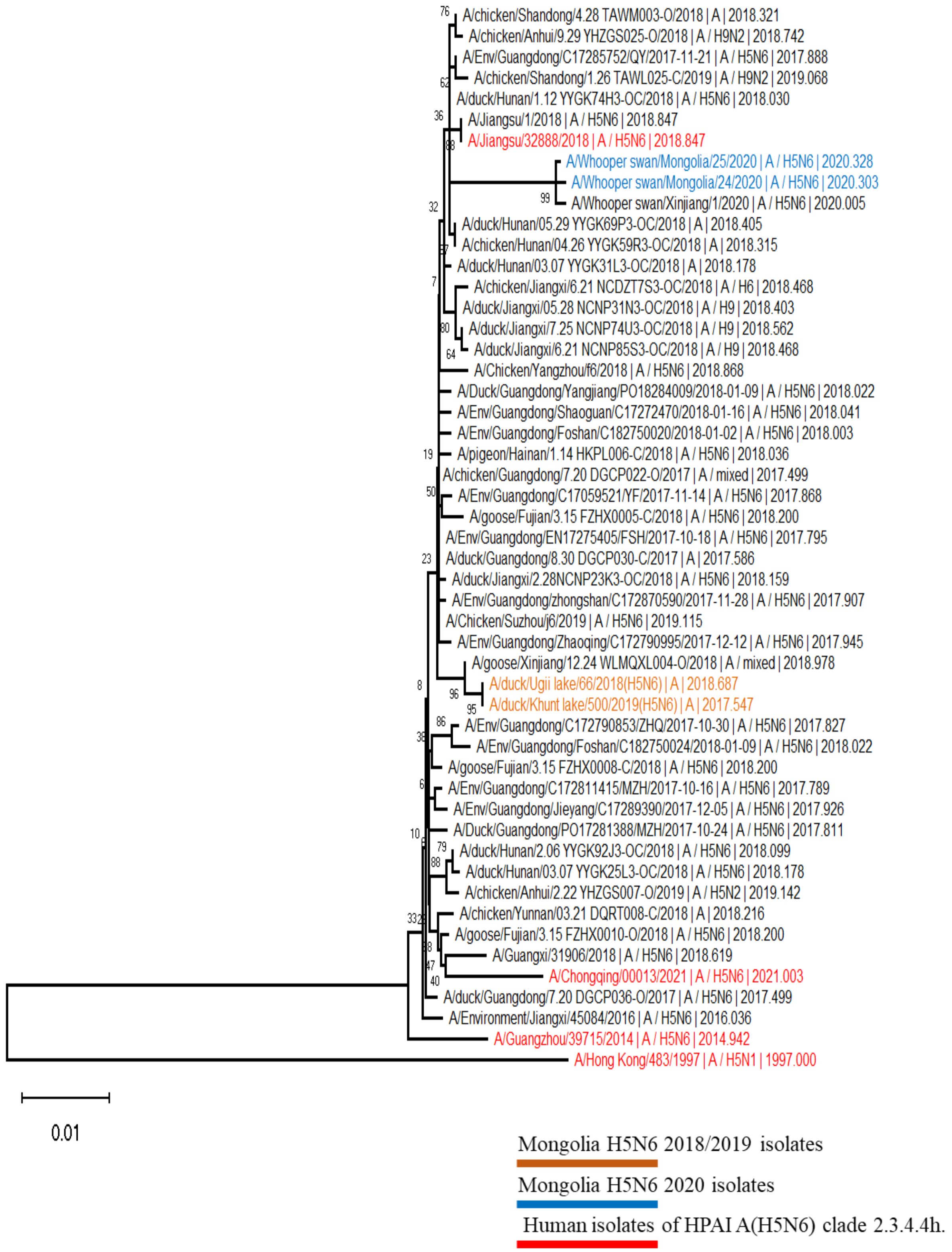

A/chicken/Shandong/4.28 TAWM003-O/2018 | A | 2018.321
A/chicken/Anhui/9.29 YHZGS025-O/2018 | A / H9N2 | 2018.742
A/Env/Guangdong/C17285752/QY/2017-11-21 | A / H5N6 | 2017.888
A/chicken/Shandong/1.26 TAWL025-C/2019 | A / H9N2 | 2019.068
A/duck/Hunan/1.12 YYGK74H3-OC/2018 | A / H5N6 | 2018.030
A/Jiangsu/1/2018 | A / H5N6 | 2018.847
A/Jiangsu/32888/2018 | A / H5N6 | 2018.847
A/Whooper swan/Mongolia/25/2020 | A / H5N6 | 2020.328
A/Whooper swan/Mongolia/24/2020 | A / H5N6 | 2020.303
A/Whooper swan/Xinjiang/1/2020 | A / H5N6 | 2020.005
A/duck/Hunan/05.29 YYGK69P3-OC/2018 | A | 2018.405
A/chicken/Hunan/04.26 YYGK59R3-OC/2018 | A | 2018.315
A/duck/Hunan/03.07 YYGK31L3-OC/2018 | A | 2018.178
A/chicken/Jiangxi/6.21 NCDZT7S3-OC/2018 | A / H6 | 2018.468
A/duck/Jiangxi/05.28 NCNP31N3-OC/2018 | A / H9 | 2018.403
A/duck/Jiangxi/7.25 NCNP74U3-OC/2018 | A / H9 | 2018.562
A/duck/Jiangxi/6.21 NCNP85S3-OC/2018 | A / H9 | 2018.468
A/Chicken/Yangzhou/f6/2018 | A / H5N6 | 2018.868
A/Duck/Guangdong/Yangjiang/PO18284009/2018-01-09 | A / H5N6 | 2018.022
A/Env/Guangdong/Shaoguan/C17272470/2018-01-16 | A / H5N6 | 2018.041
A/Env/Guangdong/Foshan/C182750020/2018-01-02 | A / H5N6 | 2018.003
A/pigeon/Hainan/1.14 HKPL006-C/2018 | A / H5N6 | 2018.036
A/chicken/Guangdong/7.20 DGCP022-O/2017 | A / mixed | 2017.499
A/Env/Guangdong/C17059521/YF/2017-11-14 | A / H5N6 | 2017.868
A/goose/Fujian/3.15 FZHX0005-C/2018 | A / H5N6 | 2018.200
A/Env/Guangdong/EN17275405/FSH/2017-10-18 | A / H5N6 | 2017.795
A/duck/Guangdong/8.30 DGCP030-C/2017 | A | 2017.586
A/duck/Jiangxi/2.28NCNP23K3-OC/2018 | A / H5N6 | 2018.159
A/Env/Guangdong/zhongshan/C172870590/2017-11-28 | A / H5N6 | 2017.907
A/Chicken/Suzhou/j6/2019 | A / H5N6 | 2019.115
A/Env/Guangdong/Zhaoqing/C172790995/2017-12-12 | A / H5N6 | 2017.945
A/goose/Xinjiang/12.24 WLMQXL004-O/2018 | A / mixed | 2018.978
A/duck/Ugii lake/66/2018(H5N6) | A | 2018.687
A/duck/Khunt lake/500/2019(H5N6) | A | 2017.547
A/Env/Guangdong/C172790853/ZHQ/2017-10-30 | A / H5N6 | 2017.827
A/Env/Guangdong/Foshan/C182750024/2018-01-09 | A / H5N6 | 2018.022
A/goose/Fujian/3.15 FZHX0008-C/2018 | A / H5N6 | 2018.200
A/Env/Guangdong/C172811415/MZH/2017-10-16 | A / H5N6 | 2017.789
A/Env/Guangdong/Jieyang/C17289390/2017-12-05 | A / H5N6 | 2017.926
A/Duck/Guangdong/PO17281388/MZH/2017-10-24 | A / H5N6 | 2017.811
A/duck/Hunan/2.06 YYGK92J3-OC/2018 | A / H5N6 | 2018.099
A/duck/Hunan/03.07 YYGK25L3-OC/2018 | A / H5N6 | 2018.178
A/chicken/Anhui/2.22 YHZGS007-O/2019 | A / H5N2 | 2019.142
A/chicken/Yunnan/03.21 DQRT008-C/2018 | A | 2018.216
A/goose/Fujian/3.15 FZHX0010-O/2018 | A / H5N6 | 2018.200
A/Guangxi/31906/2018 | A / H5N6 | 2018.619
A/Chongqing/00013/2021 | A / H5N6 | 2021.003
A/duck/Guangdong/7.20 DGCP036-O/2017 | A / H5N6 | 2017.499
A/Environment/Jiangxi/45084/2016 | A / H5N6 | 2016.036
A/Guangzhou/39715/2014 | A / H5N6 | 2014.942
A/Hong Kong/483/1997 | A / H5N1 | 1997.000
0.01
Mongolia H5N6 2018/2019 isolates
Mongolia H5N6 2020 isolates
Human isolates of HPAI A(H5N6) clade 2.3.4.4h.

F (NA)

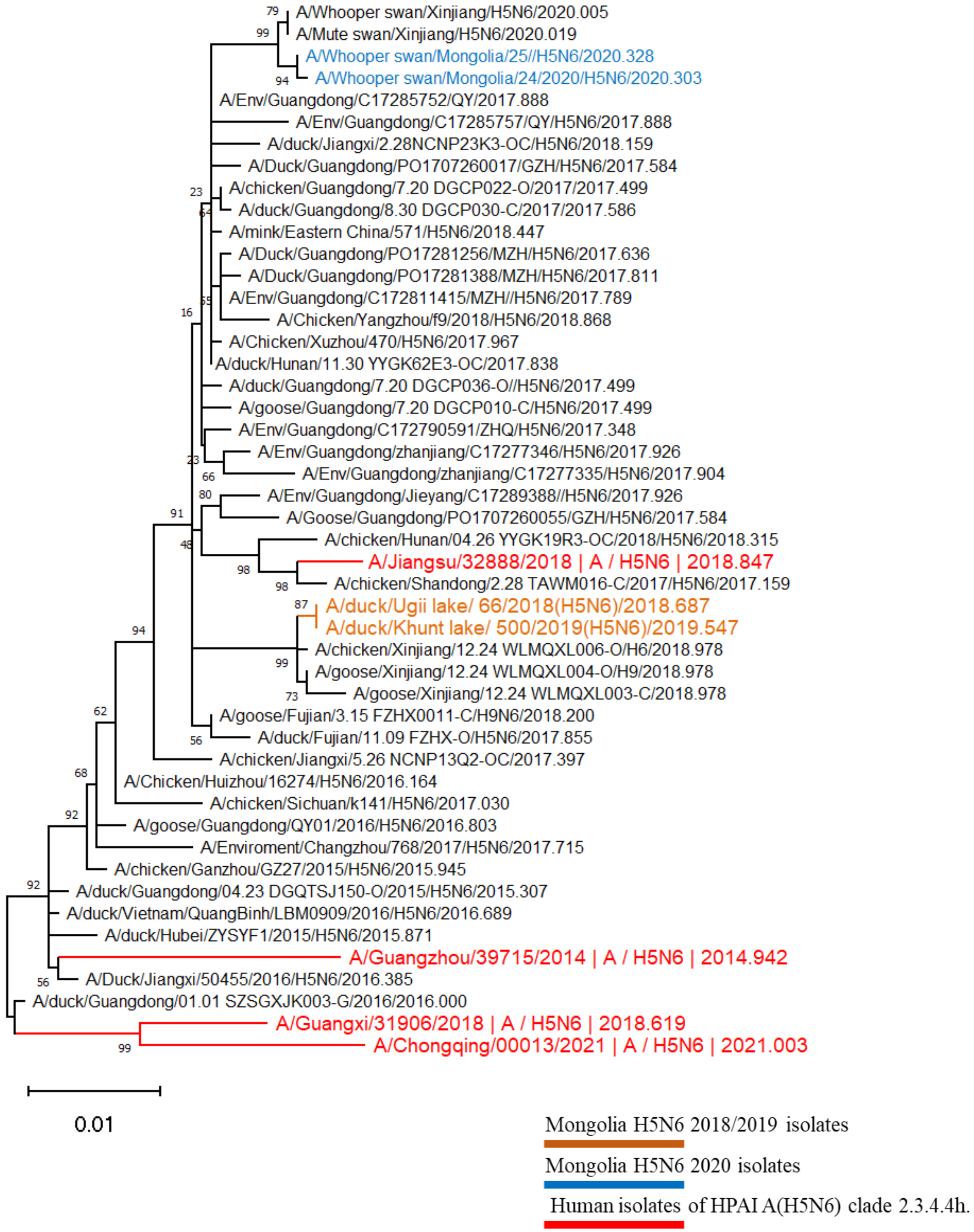
A/Whooper swan/Xinjiang/H5N6/2020.005
A/Mute swan/Xinjiang/H5N6/2020.019
A/Whooper swan/Mongolia/25//H5N6/2020.328
A/Whooper swan/Mongolia/24/2020/H5N6/2020.303
A/Env/Guangdong/C17285752/QY/2017.888
A/Env/Guangdong/C17285757/QY/H5N6/2017.888
A/duck/Jiangxi/2.28NCNP23K3-OC/H5N6/2018.159
A/Duck/Guangdong/PO1707260017/GZH/H5N6/2017.584
A/chicken/Guangdong/7.20 DGCP022-O/2017/2017.499
A/duck/Guangdong/8.30 DGCP030-C/2017/2017.586
A/mink/Eastern China/571/H5N6/2018.447
A/Duck/Guangdong/PO17281256/MZH/H5N6/2017.636
A/Duck/Guangdong/PO17281388/MZH/H5N6/2017.811
A/Env/Guangdong/C172811415/MZH//H5N6/2017.789
A/Chicken/Yangzhou/f9/2018/H5N6/2018.868
A/Chicken/Xuzhou/470/H5N6/2017.967
A/duck/Hunan/11.30 YYGK62E3-OC/2017.838
A/duck/Guangdong/7.20 DGCP036-O//H5N6/2017.499
A/goose/Guangdong/7.20 DGCP010-C/H5N6/2017.499
A/Env/Guangdong/C172790591/ZHQ/H5N6/2017.348
A/Env/Guangdong/zhanjiang/C17277346/H5N6/2017.926
A/Env/Guangdong/zhanjiang/C17277335/H5N6/2017.904
A/Env/Guangdong/Jieyang/C17289388//H5N6/2017.926
A/Goose/Guangdong/PO1707260055/GZH/H5N6/2017.584
A/chicken/Hunan/04.26 YYGK19R3-OC/2018/H5N6/2018.315
A/Jiangsu/32888/2018 | A / H5N6 | 2018.847
A/chicken/Shandong/2.28 TAWM016-C/2017/H5N6/2017.159
A/duck/Ugii lake/ 66/2018(H5N6)/2018.687
A/duck/Khunt lake/ 500/2019(H5N6)/2019.547
A/chicken/Xinjiang/12.24 WLMQXL006-O/H6/2018.978
A/goose/Xinjiang/12.24 WLMQXL004-O/H9/2018.978
A/goose/Xinjiang/12.24 WLMQXL003-C/2018.978
A/goose/Fujian/3.15 FZHX0011-C/H9N6/2018.200
A/duck/Fujian/11.09 FZHX-O/H5N6/2017.855
A/chicken/Jiangxi/5.26 NCNP13Q2-OC/2017.397
A/Chicken/Huizhou/16274/H5N6/2016.164
A/chicken/Sichuan/k141/H5N6/2017.030
A/goose/Guangdong/QY01/2016/H5N6/2016.803
A/Enviroment/Changzhou/768/2017/H5N6/2017.715
A/chicken/Ganzhou/GZ27/2015/H5N6/2015.945
A/duck/Guangdong/04.23 DGQTSJ150-O/2015/H5N6/2015.307
A/duck/Vietnam/QuangBinh/LBM0909/2016/H5N6/2016.689
A/duck/Hubei/ZYSYF1/2015/H5N6/2015.871
A/Guangzhou/39715/2014 | A / H5N6 | 2014.942
A/Duck/Jiangxi/50455/2016/H5N6/2016.385
A/duck/Guangdong/01.01 SZSGXJK003-G/2016/2016.000
A/Guangxi/31906/2018 | A / H5N6 | 2018.619
A/Chongqing/00013/2021 | A / H5N6 | 2021.003
0.01
Mongolia H5N6 2018/2019 isolates
Mongolia H5N6 2020 isolates
Human isolates of HPAI A(H5N6) clade 2.3.4.4h.

G (M)

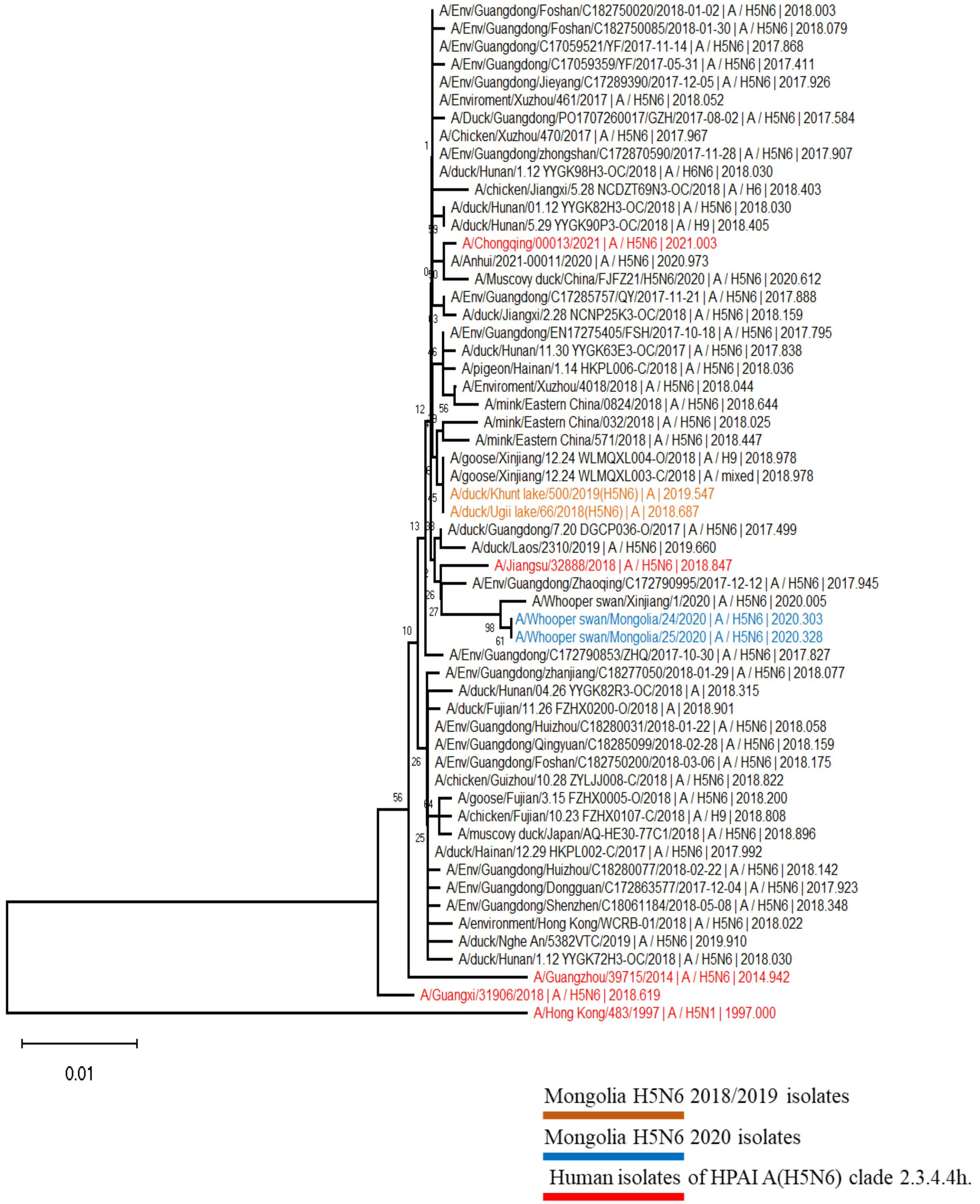

A/Env/Guangdong/Foshan/C182750020/2018-01-02 | A / H5N6 | 2018.003
A/Env/Guangdong/Foshan/C182750085/2018-01-30 | A / H5N6 | 2018.079
A/Env/Guangdong/C17059521/YF/2017-11-14 | A / H5N6 | 2017.868
A/Env/Guangdong/C17059359/YF/2017-05-31 | A / H5N6 | 2017.411
A/Env/Guangdong/Jieyang/C17289390/2017-12-05 | A / H5N6 | 2017.926
A/Enviroment/Xuzhou/461/2017 | A / H5N6 | 2018.052
A/Duck/Guangdong/PO1707260017/GZH/2017-08-02 | A / H5N6 | 2017.584
A/Chicken/Xuzhou/470/2017 | A / H5N6 | 2017.967
A/Env/Guangdong/zhongshan/C172870590/2017-11-28 | A / H5N6 | 2017.907
A/duck/Hunan/1.12 YYGK98H3-OC/2018 | A / H6N6 | 2018.030
A/chicken/Jiangxi/5.28 NCDZT69N3-OC/2018 | A / H6 | 2018.403
A/duck/Hunan/01.12 YYGK82H3-OC/2018 | A / H5N6 | 2018.030
A/duck/Hunan/5.29 YYGK90P3-OC/2018 | A / H9 | 2018.405
A/Chongqing/00013/2021 | A / H5N6 | 2021.003
A/Anhui/2021-00011/2020 | A / H5N6 | 2020.973
A/Muscovy duck/China/FJFZ21/H5N6/2020 | A / H5N6 | 2020.612
A/Env/Guangdong/C17285757/QY/2017-11-21 | A / H5N6 | 2017.888
A/duck/Jiangxi/2.28 NCNP25K3-OC/2018 | A / H5N6 | 2018.159
A/Env/Guangdong/EN17275405/FSH/2017-10-18 | A / H5N6 | 2017.795
A/duck/Hunan/11.30 YYGK63E3-OC/2017 | A / H5N6 | 2017.838
A/pigeon/Hainan/1.14 HKPL006-C/2018 | A / H5N6 | 2018.036
A/Enviroment/Xuzhou/4018/2018 | A / H5N6 | 2018.044
A/mink/Eastern China/0824/2018 | A / H5N6 | 2018.644
A/mink/Eastern China/032/2018 | A / H5N6 | 2018.025
A/mink/Eastern China/571/2018 | A / H5N6 | 2018.447
A/goose/Xinjiang/12.24 WLMQXL004-O/2018 | A / H9 | 2018.978
A/goose/Xinjiang/12.24 WLMQXL003-C/2018 | A / mixed | 2018.978
A/duck/Khunt lake/500/2019(H5N6) | A | 2019.547
A/duck/Ugii lake/66/2018(H5N6) | A | 2018.687
A/duck/Guangdong/7.20 DGCP036-O/2017 | A / H5N6 | 2017.499
A/duck/Laos/2310/2019 | A / H5N6 | 2019.660
A/Jiangsu/32888/2018 | A / H5N6 | 2018.847
A/Env/Guangdong/Zhaoqing/C172790995/2017-12-12 | A / H5N6 | 2017.945
A/Whooper swan/Xinjiang/1/2020 | A / H5N6 | 2020.005
A/Whooper swan/Mongolia/24/2020 | A / H5N6 | 2020.303
A/Whooper swan/Mongolia/25/2020 | A / H5N6 | 2020.328
A/Env/Guangdong/C172790853/ZHQ/2017-10-30 | A / H5N6 | 2017.827
A/Env/Guangdong/zhanjiang/C18277050/2018-01-29 | A / H5N6 | 2018.077
A/duck/Hunan/04.26 YYGK82R3-OC/2018 | A | 2018.315
A/duck/Fujian/11.26 FZHX0200-O/2018 | A | 2018.901
A/Env/Guangdong/Huizhou/C18280031/2018-01-22 | A / H5N6 | 2018.058
A/Env/Guangdong/Qingyuan/C18285099/2018-02-28 | A / H5N6 | 2018.159
A/Env/Guangdong/Foshan/C182750200/2018-03-06 | A / H5N6 | 2018.175
A/chicken/Guizhou/10.28 ZYLJJ008-C/2018 | A / H5N6 | 2018.822
A/goose/Fujian/3.15 FZHX0005-O/2018 | A / H5N6 | 2018.200
A/chicken/Fujian/10.23 FZHX0107-C/2018 | A / H9 | 2018.808
A/muscovy duck/Japan/AQ-HE30-77C1/2018 | A / H5N6 | 2018.896
A/duck/Hainan/12.29 HKPL002-C/2017 | A / H5N6 | 2017.992
A/Env/Guangdong/Huizhou/C18280077/2018-02-22 | A / H5N6 | 2018.142
A/Env/Guangdong/Dongguan/C172863577/2017-12-04 | A / H5N6 | 2017.923
A/Env/Guangdong/Shenzhen/C18061184/2018-05-08 | A / H5N6 | 2018.348
A/environment/Hong Kong/WCRB-01/2018 | A / H5N6 | 2018.022
A/duck/Nghe An/5382VTC/2019 | A / H5N6 | 2019.910
A/duck/Hunan/1.12 YYGK72H3-OC/2018 | A / H5N6 | 2018.030
A/Guangzhou/39715/2014 | A / H5N6 | 2014.942
A/Guangxi/31906/2018 | A / H5N6 | 2018.619
A/Hong Kong/483/1997 | A / H5N1 | 1997.000
0.01
Mongolia H5N6 2018/2019 isolates
Mongolia H5N6 2020 isolates
Human isolates of HPAI A(H5N6) clade 2.3.4.4h.

H (NS)

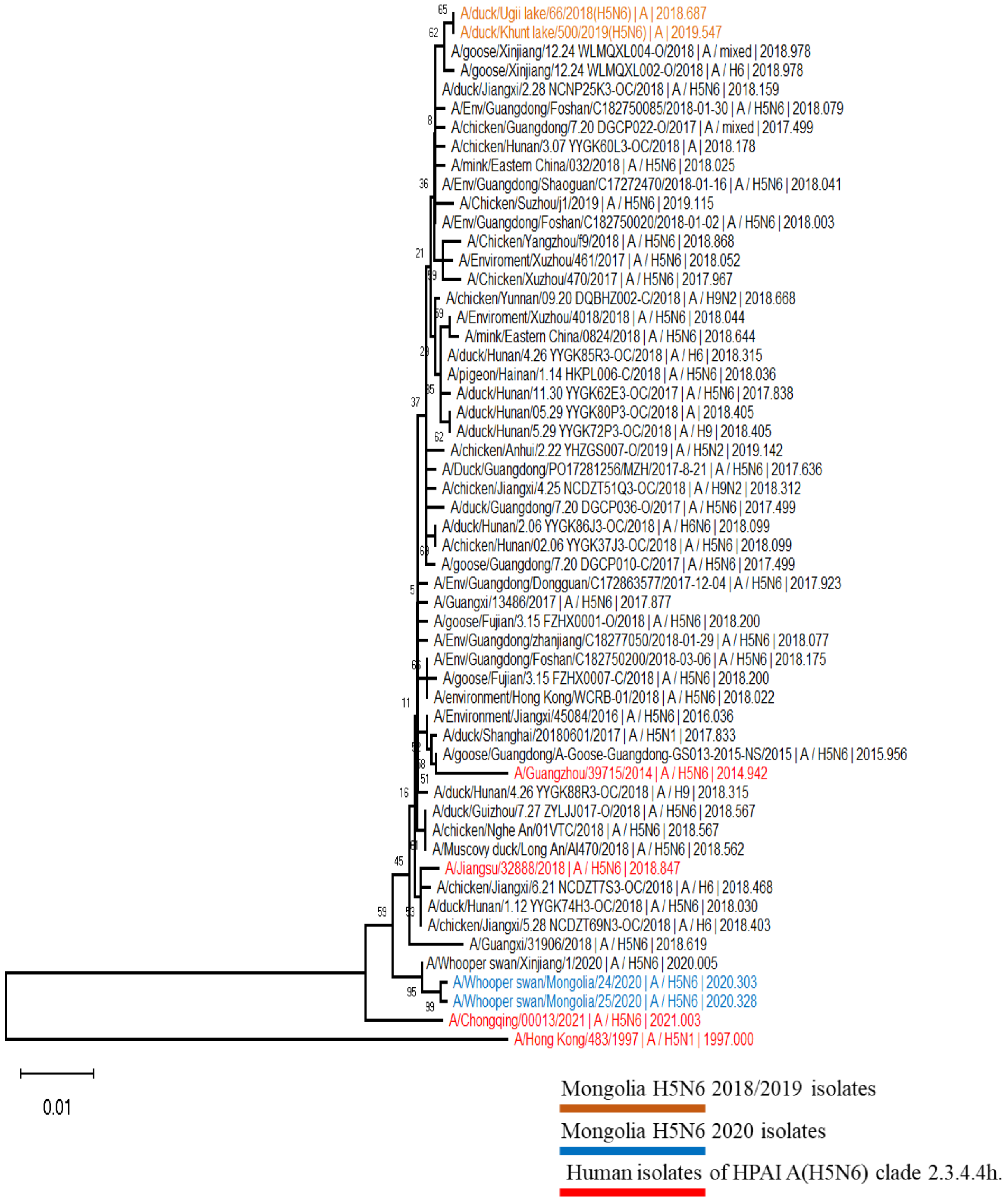
A/duck/Ugii lake/66/2018(H5N6) | A | 2018.687
A/duck/Khunt lake/500/2019(H5N6) | A | 2019.547
A/goose/Xinjiang/12.24 WLMQXL004-O/2018 | A / mixed | 2018.978
A/goose/Xinjiang/12.24 WLMQXL002-O/2018 | A / H6 | 2018.978
A/duck/Jiangxi/2.28 NCNP25K3-OC/2018 | A / H5N6 | 2018.159
A/Env/Guangdong/Foshan/C182750085/2018-01-30 | A / H5N6 | 2018.079
A/chicken/Guangdong/7.20 DGCP022-O/2017 | A / mixed | 2017.499
A/chicken/Hunan/3.07 YYGK60L3-OC/2018 | A | 2018.178
A/mink/Eastern China/032/2018 | A / H5N6 | 2018.025
A/Env/Guangdong/Shaoguan/C17272470/2018-01-16 | A / H5N6 | 2018.041
A/Chicken/Suzhou/j1/2019 | A / H5N6 | 2019.115
A/Env/Guangdong/Foshan/C182750020/2018-01-02 | A / H5N6 | 2018.003
A/Chicken/Yangzhou/f9/2018 | A / H5N6 | 2018.868
A/Enviroment/Xuzhou/461/2017 | A / H5N6 | 2018.052
A/Chicken/Xuzhou/470/2017 | A / H5N6 | 2017.967
A/chicken/Yunnan/09.20 DQBHZ002-C/2018 | A / H9N2 | 2018.668
A/Enviroment/Xuzhou/4018/2018 | A / H5N6 | 2018.044
A/mink/Eastern China/0824/2018 | A / H5N6 | 2018.644
A/duck/Hunan/4.26 YYGK85R3-OC/2018 | A / H6 | 2018.315
A/pigeon/Hainan/1.14 HKPL006-C/2018 | A / H5N6 | 2018.036
A/duck/Hunan/11.30 YYGK62E3-OC/2017 | A / H5N6 | 2017.838
A/duck/Hunan/05.29 YYGK80P3-OC/2018 | A | 2018.405
A/duck/Hunan/5.29 YYGK72P3-OC/2018 | A / H9 | 2018.405
A/chicken/Anhui/2.22 YHZGS007-O/2019 | A / H5N2 | 2019.142
A/Duck/Guangdong/PO17281256/MZH/2017-8-21 | A / H5N6 | 2017.636
A/chicken/Jiangxi/4.25 NCDZT51Q3-OC/2018 | A / H9N2 | 2018.312
A/duck/Guangdong/7.20 DGCP036-O/2017 | A / H5N6 | 2017.499
A/duck/Hunan/2.06 YYGK86J3-OC/2018 | A / H6N6 | 2018.099
A/chicken/Hunan/02.06 YYGK37J3-OC/2018 | A / H5N6 | 2018.099
A/goose/Guangdong/7.20 DGCP010-C/2017 | A / H5N6 | 2017.499
A/Env/Guangdong/Dongguan/C172863577/2017-12-04 | A / H5N6 | 2017.923
A/Guangxi/13486/2017 | A / H5N6 | 2017.877
A/goose/Fujian/3.15 FZHX0001-O/2018 | A / H5N6 | 2018.200
A/Env/Guangdong/zhanjiang/C18277050/2018-01-29 | A / H5N6 | 2018.077
A/Env/Guangdong/Foshan/C182750200/2018-03-06 | A / H5N6 | 2018.175
A/goose/Fujian/3.15 FZHX0007-C/2018 | A / H5N6 | 2018.200
A/environment/Hong Kong/WCRB-01/2018 | A / H5N6 | 2018.022
A/Environment/Jiangxi/45084/2016 | A / H5N6 | 2016.036
A/duck/Shanghai/20180601/2017 | A / H5N1 | 2017.833
A/goose/Guangdong/A-Goose-Guangdong-GS013-2015-NS/2015 | A / H5N6 | 2015.956
A/Guangzhou/39715/2014 | A / H5N6 | 2014.942
A/duck/Hunan/4.26 YYGK88R3-OC/2018 | A / H9 | 2018.315
A/duck/Guizhou/7.27 ZYLJJ017-O/2018 | A / H5N6 | 2018.567
A/chicken/Nghe An/01VTC/2018 | A / H5N6 | 2018.567
A/Muscovy duck/Long An/AI470/2018 | A / H5N6 | 2018.562
A/Jiangsu/32888/2018 | A / H5N6 | 2018.847
A/chicken/Jiangxi/6.21 NCDZT7S3-OC/2018 | A / H6 | 2018.468
A/duck/Hunan/1.12 YYGK74H3-OC/2018 | A / H5N6 | 2018.030
A/chicken/Jiangxi/5.28 NCDZT69N3-OC/2018 | A / H6 | 2018.403
A/Guangxi/31906/2018 | A / H5N6 | 2018.619
A/Whooper swan/Xinjiang/1/2020 | A / H5N6 | 2020.005
A/Whooper swan/Mongolia/24/2020 | A / H5N6 | 2020.303
A/Whooper swan/Mongolia/25/2020 | A / H5N6 | 2020.328
A/Chongqing/00013/2021 | A / H5N6 | 2021.003
A/Hong Kong/483/1997 | A / H5N1 | 1997.000
0.01
Mongolia H5N6 2018/2019 isolates
Mongolia H5N6 2020 isolates
Human isolates of HPAI A(H5N6) clade 2.3.4.4h.

Appendix Figure 1. Phylogenetic analysis of A/MN/H5N6/2018-19 isolates. Phylogenetic trees for each of the eight gene segments were constructed using MEGA 11 (Molecular Evolutionary Genetics Analysis version 11, Pennsylvania State University, PA, USA) and the neighbor joining method with a maximum composite likelihood model. A total of 1,000 bootstrap replicates were used to determine the statistical significance (https://www.megasoftware.net/) of each branch including hemagglutinin (HA) (A); neuraminidase (NA) (B); polymerase basic (PB2) (C); polymerase basic (PB1) (D); polymerase (PA) (E); nucleoprotein (NP) (F); matrix (M) (G); Nonstructural protein (NS) (H). The two HPAIVs MG/H5N6 2018/19 isolated in this study are shown in orange, the H5N6/2020 Mongolia isolate is in blue and the HPAIVs H5 human isolates are in red.
